# Complications in acute respiratory distress syndrome: a systematic review and meta-analysis

**DOI:** 10.1186/s13054-026-05978-y

**Published:** 2026-03-29

**Authors:** David Granton, Omar I. Hajjaj, Laith Ishaq, Roja Ahimsadasan, Sydni G. Paleczny, Nilah Ahimsadasan, Elliot Ying, Ghaida Jabri, Azam Mansuri, Niall Warfield, Tommaso Pettenuzzo, Kimia Honarmand, Pooja Gandhi, Marina Englesakis, Eddy Fan

**Affiliations:** 1 Interdepartmental Division of Critical Care Medicine, University of Toronto, 585 University Avenue, 9-MaRS-9013, Toronto, M5G 2N2 ON Canada; 2 Department of Medicine, Queen’s University, Kingston, Canada; 3 Schulich School of Medicine & Dentistry, Western University, London, Canada; 4 Western Institute for Neuroscience, Western University, London, Canada; 5 Queen’s School of Medicine, Kingston, Canada; 6 Faculty of Health Sciences, McMaster University, Hamilton, Canada; 7 Division of Critical Care, Department of Medicine, King Faisal Specialist Hospital and Research Center, Medina, Saudi Arabia; 8 Faculty of Health Sciences, Queen’s University, Kingston, Canada; 9 Department of Medicine, Georgetown University, Baltimore, USA; 10 Department of Medicine, Institute of Anesthesia and Intensive Care, University of Padua, Padua, Italy; 11 Division of Critical Care, Department of Medicine, McMaster University, Hamilton, Canada; 12 Department of Communication Sciences and Disorders, Faculty of Rehabilitation Medicine, University of Alberta, Edmonton, AB Canada; 13 Library & Information Services, University Health Network, Toronto, ON Canada

**Keywords:** Acute respiratory distress syndrome, Invasive mechanical ventilation, Complications

## Abstract

**Background:**

Acute respiratory distress syndrome (ARDS) carries substantial morbidity and mortality. Supportive care with invasive mechanical ventilation (IMV) remains a cornerstone of management. The breadth and prevalence of complications experienced by patients with ARDS undergoing IMV is unclear.

**Methods:**

We performed a systematic review and meta-analysis to quantify complications reported by studies featuring patients with ARDS undergoing IMV. We searched MEDLINE, MEDLINE In-Process/ePubs, EMBASE, Cochrane Central Register of Controlled Trials, Cochrane Database of Systematic Reviews and ClinicalTrials.gov from database inception until November 28, 2025. We included randomized controlled trials (RCTs) and cohort studies featuring adults with ARDS undergoing IMV that reported at least one complication. We excluded studies of COVID-19, studies with 25% or more patients on extracorporeal life support, and studies with under 200 participants. We extracted data on any reported clinical complication, based on author definitions. For complications reported by three or more studies we performed a random effects meta-analysis of logit-transformed complication proportions using inverse-variance weighting.

**Results:**

Of 25,421 citations, we reviewed 2620 full texts and included 53 studies (25 RCTs and 28 cohort studies). Complications were variably and infrequently reported. Only barotrauma, ventilator associated pneumonia (VAP), hypotension, arrhythmia, stroke, myopathy and cardiac arrest were reported by three or more RCTs. In cohort studies, barotrauma, VAP, acute renal failure, sepsis and bacteremia were reported by three or more studies. All estimates featured considerable heterogeneity.

**Conclusions:**

In this systematic review of studies including patients with ARDS receiving IMV, reporting of complications was variable and infrequent. Our synthesis was descriptive and did not investigate causality. Future studies should establish consensus on the spectrum of complications to improve reporting and help evaluate the risks and benefits of novel therapies. We propose a process to develop a core outcome set for complications experienced by patients with ARDS undergoing IMV. Preliminary results of this work were presented at the ESICM LIVES conference in October 2021

**Supplementary Information:**

The online version contains supplementary material available at 10.1186/s13054-026-05978-y.

## Background

Acute respiratory distress syndrome (ARDS) is a heterogenous syndrome of acute respiratory failure secondary to pulmonary inflammation not explained by cardiac failure [1]. The mainstay of management is largely supportive with lung protective ventilation in those undergoing invasive mechanical ventilation (IMV) [1–6]. Although non-invasive oxygenation strategies can be utilized in mild disease, most patients with ARDS require IMV. A large, international observational study estimated IMV usage in ARDS at approximately 90% [1, 7].

While lifesaving, the use of IMV is not without risk. Direct pulmonary injury related to IMV, termed ventilator-induced lung injury (VILI), is a well described phenomenon [8, 9]. VILI includes grossly apparent injury like barotrauma and more subtle injury, such as overdistention (volutrauma) and repetitive opening and closing of unstable lung units (atelectrauma). These mechanical forces imposed by IMV on pulmonary tissue may result in release of systemic inflammatory mediators that contribute to multiorgan dysfunction and death [8, 10]. Limiting tidal volume, plateau pressure and driving pressure has been associated with reduced mortality, highlighting the importance of VILI and risks of IMV [2, 11, 12].

Complications experienced by patients with ARDS receiving IMV extend beyond direct pulmonary injury. For example, cardiopulmonary interactions during IMV can result in right ventricular strain and hypotension, potentially contributing to the development of renal failure [13, 14]. Additionally, these patients can develop a systemic inflammatory response with resultant multiorgan dysfunction [10, 15]. Lastly, nosocomial complications are common in the ICU and can have a devastating impact on patient morbidity and mortality [16]. To our knowledge, no report exists that systematically quantifies the range and frequency of complications in individuals with ARDS receiving IMV. This is an important knowledge gap as thoroughly understanding the range of complications experienced by patients with ARDS undergoing IMV can help inform decision making. For example, understanding baseline risks is important when considering if alternative proposed therapies (e.g. extracorporeal support) offer an improved risk/benefit profile. To provide evidence on the diversity and frequency of complications experienced by patients with ARDS undergoing IMV, we conducted a systematic review and meta-analysis of studies with ARDS patients receiving IMV that reported complications.

## Methods

This review was reported in accordance with the Preferred Reporting Items for Systematic Reviews and Meta-Analyses (PRISMA) recommendations [17] and pre-registered on PROSPERO (CRD42020161960).

### Data sources and searches

We performed a systematic search using six databases (MEDLINE, MEDLINE In-Process/ePubs, EMBASE, Cochrane Central Register of Controlled Trials, Cochrane Database of Systematic Reviews and ClinicalTrials.gov). Our initial search was on September 7, 2019 and included any citations meeting criteria from the inception of each database. Our search was updated on August 17, 2023 and November 28th, 2025. We searched for studies of mechanically ventilated adults with ARDS using keywords “Acute Respiratory Distress Syndrome” together with “Mechanical Ventilation” and “Complications” or “Harm” or “Etiology”. We excluded non-English literature and conference abstracts. (Additional File 1: Search Strategy).

### Study selection

Reviewers (DG, OH, LI, RA, SP, NA, EY, GJ, AM, NW, TP, KH, PG) screened citations independently and in duplicate. We reviewed citations by title and abstract, and then by full text for any identified as potentially relevant. Disagreements at full text review were resolved by adjudication (DG, LI, or EF) or discussion. For the initial search (up to September 7, 2019) screening was performed using Distiller SR. The updated search (up to August 17, 2023) and data extraction utilized Covidence.

We included randomized controlled trials (RCTs) and retrospective or prospective cohort studies with adult patients diagnosed with ARDS undergoing IMV that reported at least one complication. This did not include outcomes such as mortality or length of stay. We did not include “days of” organ system dysfunction to represent a clinical complication or make assumptions of complication occurrence based on reported physiologic or laboratory values. We screened supplements of all potentially eligible studies for complications. Post-hoc, we excluded studies with 200 or fewer participants to focus on larger reports more likely to report complications.

We excluded reviews, case reports, case-control studies, editorials, letters, cross sectional studies, and abstracts. To avoid duplicate reporting, we excluded studies that included participants described in previously published literature unless the initial study did not meet inclusion criteria. For studies that used multi-publication cohorts or publicly available databases that sampled overlapping time periods, we included the study with most participants. We excluded studies that used administrative databases which relied solely on international classification of disease codes for identifying ARDS and complications.

We excluded animal studies and studies with pediatric participants as defined by study authors. We excluded studies that featured exclusively those who survived or died to avoid biased estimates of complications. We excluded studies that featured over 25% of participants on extracorporeal life support (ECLS) [18] to avoid reporting ECLS-related complications [19]. The COVID-19 pandemic emerged during the review, and we excluded studies that reported on patients with COVID-19.

### Data extraction

Reviewers (DG, OH, LI, RA, NA, SP, EY, AM, NW, TP, GJ) performed data abstraction independently and in duplicate. Any disagreements were resolved by adjudication (DG, LI or EF) or discussion. We abstracted study details (funding, database used etc.), inclusion and exclusion criteria, definition of ARDS, participant details (e.g., age, etiology of ARDS, comorbidities, baseline PaO_2_:FiO_2_ ratio, etc.), number of patients requiring ECLS, complications, and mortality.

### Quality assessment

To evaluate study quality for RCTs we used the Cochrane Risk of Bias (RoB-2) tool, and for cohort studies we adapted the Newcastle-Ottawa Scale (NOS) [20] (Additional File 2: eFigure 1). Studies with one star less than the possible maximum were rated as “some concerns”, while studies with two or more stars less than the possible maximum were rated as “high concern”. We considered a low-quality study to be an RCT rated as either “some concerns” or “high risk of bias” or a cohort study missing at least one star.

### Data analysis

We performed all analyses separately for RCTs and cohort studies, and all analyses were conducted in R (version 4.5.2) [21]. We summarized study-level patient demographic data, etiology of ARDS and comorbidities using descriptive statistics. For any complication reported by three or more studies, we performed a random-effects meta-analysis of proportions. Analyses were conducted on a logit-transformed proportion scale for variance stabilization. A continuity correction of 0.5 was applied for studies with zero events. We used inverse-variance weighting with between study heterogeneity estimated using restricted maximum likelihood. Confidence intervals for estimates were calculated with the Hartung-Knapp-Sidik-Jonkman method. Pooled estimates and intervals were back transformed using inverse logit. Pre-specified subgroup analyses included: studies published before versus after the year 2000, pulmonary versus extrapulmonary ARDS, severity of ARDS, study intervention, and high versus low quality studies. Given lack of granularity and heterogeneity of included studies we were unable to perform our pre-specified subgroup analyses except for high versus low study quality. For complications with at least three studies in each quality category, we fitted separate random-effects models within each subgroup, and subgroup differences were tested using meta-regression with risk of bias as a moderator.

## Results

Of 25,421 citations, we reviewed 2620 full texts and included 53 studies (Additional File 2: eFigure 2). We included 25 RCTs (*n* = 13,490) and 28 cohort studies (*n* = 23,937) (Additional File 2: eTables 1 and 2). Four RCTs were published in 2020 onwards, 13 from 2010 to 2019, seven from 2000 to 2009, and one prior to 2000. Seventeen cohort studies were published in 2020 onwards, 10 from 2010 to 2019, and one from 2000 to 2009. Seventeen RCTs and six cohort studies used the American-European Consensus Conference (AECC) definition [3] for ARDS, six RCTs and 19 cohort studies used the Berlin definition [5], and the remaining studies used other definitions or did not describe their definition. Median age was 55.4 years ((interquartile range (IQR) 51.5–58) in RCTs and 59.8 years (IQR 56-62.5) in cohort studies. Median PaO2:FiO2 ratio at baseline was 135.5 (IQR 117.9-146.7) in RCTs (23 RCTs, *n* = 12,850) and 112.3 (IQR 106-123.9) in cohort studies (13 studies, *n* = 5879). The etiology of ARDS was reported in all RCTs and in 21 of 28 cohort studies. Etiology of ARDS and patient comorbidities are summarized in the supplement (Additional File 2: eTables 3 to 8).

### Quality assessment

Two RCTs were rated as high RoB, six were rated as some concerns, and the remaining 17 were rated as low RoB (Additional File 2: eTable 9). Four cohort studies were rated as high concerns, seven cohort studies were rated as some concerns and the remaining 17 as low concerns (Additional File 2: eTables 10 and 11).

### Complications

Complications were variably and inconsistently reported across studies. In RCTs, only barotrauma, ventilator associated pneumonia (VAP), hypotension, arrhythmia, stroke, myopathy and cardiac arrest were reported by three or more RCTs (Table 1). In cohort studies, barotrauma, VAP, acute renal failure, sepsis and bacteremia were reported by three or more studies (Table 2). All estimates featured considerable heterogeneity, with resultant wide confidence and prediction intervals. Forest plots for pooled complications can be found in the supplement (Additional File 2: eFigure 3–16). Complications that were reported by less than three studies and not pooled can be found in the supplement (Additional File 2: eTables 12 and 13).

**Table 1:** Pooled prevalence of complications reported in randomized controlled trials

Complication	No. studies	Crude frequency *n*/*N* (%)	Pooled proportion (95% CI)	95% prediction interval	Heterogeneity (τ²)	Heterogeneity (I^2^, %)
Barotrauma	18	844/10,106 (8)	8% (6–10)	3–21	0.28	91.9
Ventilator associated pneumonia	7	346/3994 (9)	8% (2–31)	0–84	2.37	99.0
Arrhythmia	7	520/3485 (15)	13% (8–20)	3–38	0.3	94.2
Hypotension	3	335/1931 (17)	12% (0–98)	0–100	5.07	99.5
Stroke	3	17/1952 (0.9)	1% (0–12)	0–51	0.8	78.8
Myopathy	3	163/1991 (8)	4% (0–99)	0–100	10.63	99.7
Cardiac arrest	3	68/1676 (4)	4% (0–62)	0–98	2.12	97.0

**Table 2:** Pooled prevalence of complications reported in cohort studies

Complication	No. studies	Crude frequency *n*/*N* (%)	Pooled proportion (95% CI)	95% prediction interval	Heterogeneity (τ²)	Heterogeneity (I^2^, %)
Ventilator associated pneumonia	12	2247/11,666 (19)	22% (9–43)	2–83	1.45	99.4
Acute renal failure	10	3833/9081 (42)	48% (35–61)	14–84	0.53	99.0
Barotrauma	8	307/4028 (7)	8% (6–10)	3–17	0.13	79.1
Sepsis	5	1839/4341 (42)	44% (20–72)	4–93	0.88	99.3
Bacteremia	4	124/2034 (6)	8% (2–24)	1–58	0.6	94.3

### Mortality

Mortality was frequently reported across included studies, but the timing varied. Hospital mortality was pooled as it was reported most often overall. In RCTs, hospital mortality was 37% (95% confidence interval (CI) 30% to 44%, *n* = 12 studies) and 44% (95% CI 33% to 44%, *n* = 16 studies) in cohort studies (Additional File 2: eFigures 8 and 16).

### Subgroup analysis

Complications amenable to subgroup analyses for RCTs included barotrauma, VAP, and arrhythmia. For cohort studies, amenable complications included barotrauma, VAP, and acute renal failure. There was no evidence of a difference in pooled prevalence for any of these complications between low and high study quality (Additional File 2: Supplementary Results).

## Discussion

Our systematic review and meta-analysis identified 25 RCTs (*n* = 13,490) and 28 cohort studies (*n* = 23,937) featuring patients with ARDS receiving IMV that reported at least one complication. To our knowledge, this work provides the most comprehensive synthesis of available evidence in effort to understand the breadth and magnitude of complications experienced by patients with ARDS undergoing IMV. However, despite an extensive and systematic search, the reporting of complications was relatively infrequent. In RCTs only barotrauma, VAP, hypotension, arrhythmia, stroke, myopathy and cardiac arrest were reported in three or more studies and subsequently pooled. In cohort studies barotrauma, VAP, acute renal failure, sepsis and bacteremia were pooled. Owing to infrequent reporting and major methodological and clinical heterogeneity in included studies, all pooled estimates featured substantial heterogeneity. This limits the ability to draw any inference or conclusions on the spectrum and prevalence of complications experienced in this population. While there was no evidence of subgroup effects when analyzing eligible complications based on high or low study quality, this may reflect the substantial between-study heterogeneity and limited number of eligible studies with resultant loss of statistical power. Importantly, the complications summarized cannot be casually ascribed to IMV or ARDS, especially in the absence of patient-level data adjusting for illness severity, organ failure, and duration of ventilation [8, 10, 13–15].

The Consolidated Standards of Reporting Trials (CONSORT) statement first emphasized the importance of reporting complications in RCTs in 2001. In 2004, an extension was published that provided more detailed guidance on reporting complications, emphasizing accurate reporting of how complications are collected, defined, and reported [22]. While the Strengthening the Reporting of Observational Studies in Epidemiology (STROBE) statement (introduced in 2007) requires clear definitions and reporting of study outcomes, they do not mandate universal reporting of complications [23]. The lack of a specific guidance document outlining proper reporting of complications in observational literature may explain some of the inconsistent and heterogenous reporting of complications we saw. Other potential explanations include the lack of standardized definitions for complications in this population, the burden of additional data collection, the potential need for non-routine diagnostic testing to detect certain complications, and historical practices of focusing on metrics such as mortality and physiologic parameters in this patient population.

**Fig. 1:**
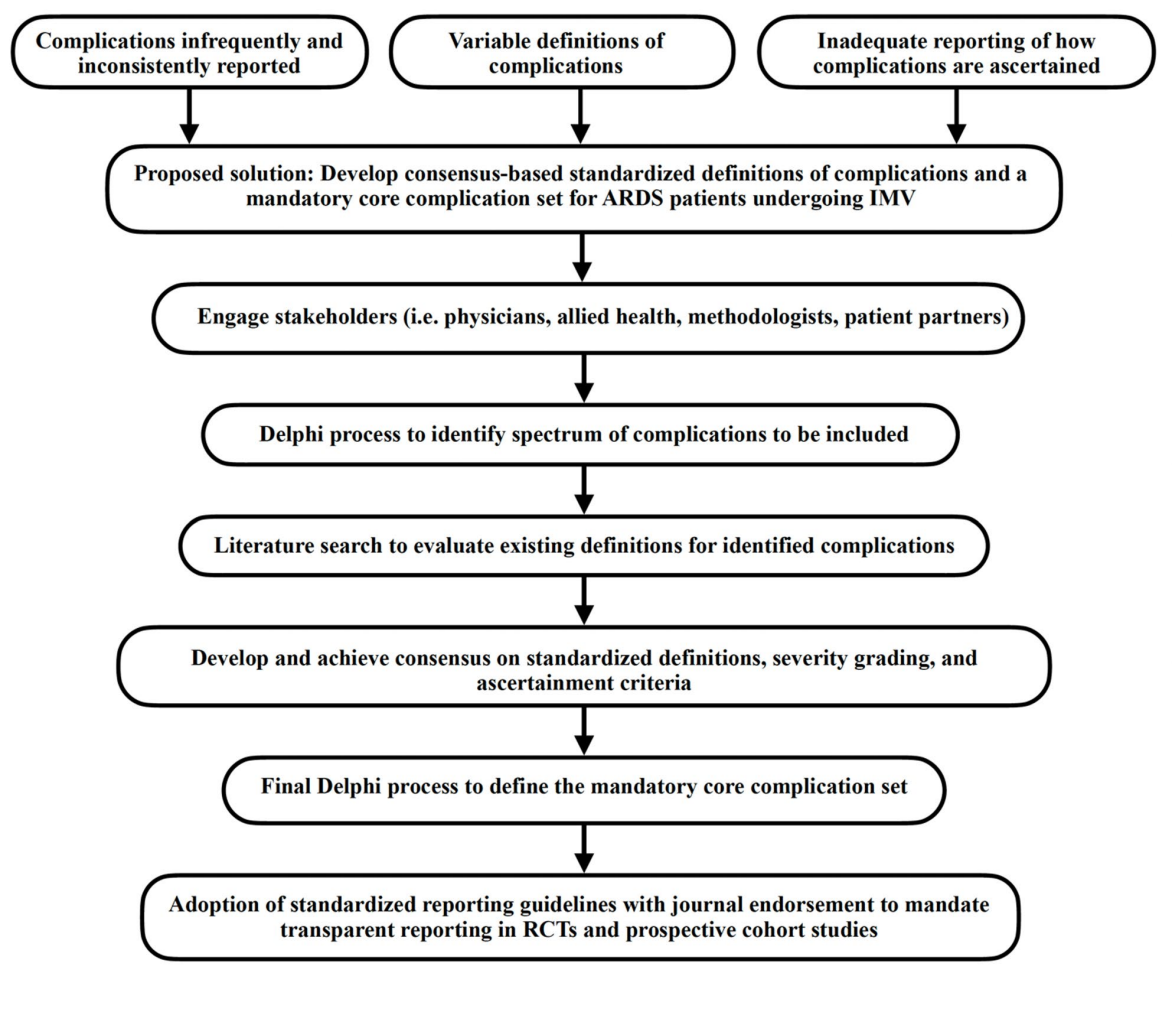
Proposed solution to standardize reporting of complications in patients mechanically ventilated with ARDS. Development and reporting of the core complication set conducted in accordance with EQUATOR’s Core Outcome Set-STAndardised Protocol Items (COS-STAP) framework. ARDS: Acute respiratory distress syndrome. IMV: Invasive mechanical ventilation. RCTs: Randomized controlled trials

We advocate for standardizing the reporting of complications in studies featuring patients with ARDS undergoing IMV to improve the inconsistencies and often absent reporting of complications observed in this review. Initiatives by the Academic Research Consortium in formalizing the spectrum and definitions of various end points and complications has been performed in cardiac and pediatric ECLS literature [18, 24, 25]. We propose that a similar approach be undertaken in this population, by developing a core outcome set of complications (Fig. 1). This would allow for an improved ability to evaluate and quantify the complications experienced by patients with ARDS undergoing IMV, as well as provide a risk benchmark for comparison with novel or alternative interventions. This is crucial when evaluating the benefits and risks of alternative therapies in ARDS. For example, venovenous extracorporeal membrane oxygenation (V-V ECMO) can facilitate gas exchange to allow for lower intensity mechanical ventilation and bridge to patient recovery. Complications of V-V ECMO are well described and include bleeding, thrombosis and cannulation related complications, among others [26, 27]. Despite these complications, evidence supports the use of V-V ECMO in certain individuals with very severe ARDS [26, 28, 29]. Thus, a thorough understanding of the breadth of complications experienced by patients with ARDS undergoing IMV is crucial when deciding whether V-V ECMO is appropriate for a patient after weighing the potential risks and benefits with escalation to V-V ECMO.

Our study has several limitations. First, the complications described cannot be causally attributed to either IMV or ARDS. Second, studies not reporting a given complication could not be included which raises the possibility of selective outcome reporting where complications may be reported only when common or felt to be clinically salient in the given study, potentially biasing estimates. Third, by using an epidemiological meta-analytic framework to estimate complication prevalence, we were unable to adjust for the exposure time of duration of IMV and death as a competing event. The inability to adjust for these factors may introduce bias into our estimates. Fourth, by excluding cohorts that described survivors, we likely missed reports on long-term outcomes such as functional and neurocognitive abnormalities that are described in these cohorts [30, 31]. Fifth, we excluded studies that featured COVID-19 ARDS. This was done given the vast amount of literature on COVID-19 related ARDS, it was not feasible to include these studies with our pre-defined inclusion criteria as the COVID-19 pandemic emerged during the review. Sixth, we amended our protocol to exclude reports featuring under 200 participants reducing the number of eligible studies and our ability to capture complications reported in smaller studies. However, this exclusion was deemed necessary given the broad search strategy and inclusion of all study sizes would not have been feasible. Larger studies may provide more accurate and generalizable estimates of complications as they are more frequently multicenter, may be more likely to feature systematic outcome reporting, and are less likely to report zero events for outcomes which avoids bias from frequent continuity correction implementation. Lastly, there was significant clinical and methodological heterogeneity across included studies. We relied on author reported complications without pre-specifying specific complication definitions to satisfy inclusion. We also allowed different ARDS definitions. This contributes substantially to heterogeneity of our estimates. However, this was done as complications and ARDS diagnostic criteria have variable definitions in the literature which change over time, and many studies did not report specific complication definitions.

## Conclusions

In this systematic review of studies including patients with ARDS receiving IMV, reporting of complications was variable and infrequent. An initiative to establish a consensus on the type and definitions of complications that should be captured in future studies of ARDS patients receiving IMV would allow for more consistent evaluation and quantification of complications experienced in this population, and provide an important risk benchmark for comparison with novel or alternative interventions.

## Supplementary Information

Below is the link to the electronic supplementary material.

Supplementary Material 1.

Supplementary Material 2.

## Data Availability

The data that support the findings of this study are available from the corresponding author upon reasonable request.

## Declarations

### Prior presentations

Interim results of this work was previously presented as a poster presentation at the ESICM LIVES conference in October 2021.

## Abbreviations

AECC: American-European Consensus Conference
ARDS: Acute respiratory distress syndrome
ECLS: Extracorporeal life support
ECMO: Extracorporeal membrane oxygenation
IMV: Invasive mechanical ventilation
NOS: Newcastle Ottawa Scale
RoB: Risk of bias
VAP: Ventilator associated pneumonia
VILI: Ventilator induced lung injury

## References

[1] Matthay MA, Arabi Y, Arroliga AC, Bernard G, Bersten AD, Brochard LJ, et al. A New Global Definition of Acute Respiratory Distress Syndrome. Am J Respir Crit Care Med. 2024;209(1):37–47. 37487152 10.1164/rccm.202303-0558WS PMC10870872

[2] Ventilation with Lower Tidal Volumes as Compared with Traditional Tidal Volumes for Acute Lung Injury and the Acute Respiratory Distress Syndrome. N Engl J Med. 2000;342(18):1301–8. 10793162 10.1056/NEJM200005043421801

[3] Bernard GR, Artigas A, Brigham KL, Carlet J, Falke K, Hudson L et al. The American-European Consensus Conference on ARDS. Definitions, mechanisms, relevant outcomes, and clinical trial coordination. American Journal of Respiratory and Critical Care Medicine. 1994;149(3):818 – 24. 10.1164/ajrccm.149.3.7509706 7509706

[4] Fernando SM, Ferreyro BL, Urner M, Munshi L, Fan E. Diagnosis and management of acute respiratory distress syndrome. Can Med Assoc J. 2021;193(21):E761–8. 34035056 10.1503/cmaj.202661 PMC8177922

[5] The ARDS Definition Task Force. Acute Respiratory Distress Syndrome: The Berlin Definition. JAMA. 2012;307(23):2526–33. 22797452 10.1001/jama.2012.5669

[6] Qadir N, Sahetya S, Munshi L, Summers C, Abrams D, Beitler J, et al. An Update on Management of Adult Patients with Acute Respiratory Distress Syndrome: An Official American Thoracic Society Clinical Practice Guideline. Am J Respir Crit Care Med. 2024;209(1):24–36. 38032683 10.1164/rccm.202311-2011ST PMC10870893

[7] Bellani G, Laffey JG, Pham T, Fan E, Brochard L, Esteban A, et al. Epidemiology, Patterns of Care, and Mortality for Patients With Acute Respiratory Distress Syndrome in Intensive Care Units in 50 Countries. JAMA. 2016;315(8):788–800. 26903337 10.1001/jama.2016.0291

[8] Slutsky AS, Ranieri VM. Ventilator-Induced Lung Injury. N Engl J Med. 2013;369(22):2126–36. 24283226 10.1056/NEJMra1208707

[9] Webb HH, Tierney DF. Experimental Pulmonary Edema due to Intermittent Positive Pressure Ventilation with High Inflation Pressures. Protection by Positive End-Expiratory Pressure. Am Rev Respir Dis. 1974;110(5):556–65. 4611290 10.1164/arrd.1974.110.5.556

[10] SLUTSKY AS, TREMBLAY LN. Multiple System Organ Failure. Am J Respir Crit Care Med. 1998;157(6):1721–5. 9620897 10.1164/ajrccm.157.6.9709092

[11] Amato MBP, Meade MO, Slutsky AS, Brochard L, Costa ELV, Schoenfeld DA, et al. Driving Pressure and Survival in the Acute Respiratory Distress Syndrome. N Engl J Med. 2015;372(8):747–55. 25693014 10.1056/NEJMsa1410639

[12] Goligher EC, Costa ELV, Yarnell CJ, Brochard LJ, Stewart TE, Tomlinson G, et al. Effect of Lowering Vt on Mortality in Acute Respiratory Distress Syndrome Varies with Respiratory System Elastance. Am J Respir Crit Care Med. 2021;203(11):1378–85. 33439781 10.1164/rccm.202009-3536OC

[13] Benites MH, Suarez-Sipmann F, Kattan E, Cruces P, Retamal J. Ventilation-induced acute kidney injury in acute respiratory failure: Do PEEP levels matter? Crit Care. 2025;29(1):130. 40114273 10.1186/s13054-025-05343-5 PMC11927345

[14] Bassi T, Taran S, Girard TD, Robba C, Goligher EC. Ventilator-associated Brain Injury: A New Priority for Research in Mechanical Ventilation. Am J Respir Crit Care Med. 2024;209(10):1186–8. 38526447 10.1164/rccm.202401-0069VP PMC11146544

[15] Jozwiak M, Teboul J-L. Heart–Lungs interactions: the basics and clinical implications. Ann Intensiv Care. 2024;14(1):122. 10.1186/s13613-024-01356-5 PMC11319696 39133379

[16] Rosenthal VD, Yin R, Nercelles P, Rivera-Molina SE, Jyoti S, Dongol R, et al. International Nosocomial Infection Control Consortium (INICC) report of health care associated infections, data summary of 45 countries for 2015 to 2020, adult and pediatric units, device-associated module. Am J Infect Control. 2024;52(9):1002–11. 38185380 10.1016/j.ajic.2023.12.019

[17] Page MJ, McKenzie JE, Bossuyt PM, Boutron I, Hoffmann TC, Mulrow CD, et al. The PRISMA 2020 statement: an updated guideline for reporting systematic reviews. BMJ. 2021;372:n71. 33782057 10.1136/bmj.n71 PMC8005924

[18] Alexander PMA, Di Nardo M, Combes A, Vogel AM, Antonini MV, Barrett N, et al. Definitions of adverse events associated with extracorporeal membrane oxygenation in children: results of an international Delphi process from the ECMO-CENTRAL ARC. Lancet Child Adolesc Health. 2024;8(10):773–80. 39299748 10.1016/S2352-4642(24)00132-9 PMC12917940

[19] Abrams D, Agerstrand C, Beitler JR, Karagiannidis C, Madahar P, Yip NH, et al. Risks and Benefits of Ultra–Lung-Protective Invasive Mechanical Ventilation Strategies with a Focus on Extracorporeal Support. Am J Respir Crit Care Med. 2022;205(8):873–82. 35044901 10.1164/rccm.202110-2252CP

[20] GA Wells BS, D O’Connell J, Peterson V, Welch M, Losos PT. The Newcastle-Ottawa Scale (NOS) for assessing the quality of nonrandomised studies in meta-analyses [Available from: https://www.ohri.ca/programs/clinical_epidemiology/oxford.asp

[21] R Core Team (2025). R: A language and environment for statistical computing. R Foundation for Statistical Computing, Vienna, Austria.

[22] Better Reporting of Harms in Randomized Trials. An Extension of the CONSORT Statement. Ann Intern Med. 2004;141(10):781–8. 15545678 10.7326/0003-4819-141-10-200411160-00009

[23] von Elm E, Altman DG, Egger M, Pocock SJ, Gøtzsche PC, Vandenbroucke JP. The Strengthening the Reporting of Observational Studies in Epidemiology (STROBE) statement: guidelines for reporting observational studies. Lancet. 2007;370(9596):1453–7. 18064739 10.1016/S0140-6736(07)61602-X

[24] Garcia-Garcia HM, McFadden EP, Farb A, Mehran R, Stone GW, Spertus J, et al. Standardized End Point Definitions for Coronary Intervention Trials: The Academic Research Consortium-2 Consensus Document. Circulation. 2018;137(24):2635–50. 29891620 10.1161/CIRCULATIONAHA.117.029289

[25] Lansky AJ, Messé SR, Brickman AM, Dwyer M, van der Worp HB, Lazar RM, et al. Proposed Standardized Neurological Endpoints for Cardiovascular Clinical Trials: An Academic Research Consortium Initiative. J Am Coll Cardiol. 2017;69(6):679–91. 28183511 10.1016/j.jacc.2016.11.045

[26] Munshi L, Walkey A, Goligher E, Pham T, Uleryk EM, Fan E. Venovenous extracorporeal membrane oxygenation for acute respiratory distress syndrome: a systematic review and meta-analysis. Lancet Respir Med. 2019;7(2):163–72. 30642776 10.1016/S2213-2600(18)30452-1

[27] Teijeiro-Paradis R, Gannon WD, Fan E. Complications Associated With Venovenous Extracorporeal Membrane Oxygenation-What Can Go Wrong? Crit Care Med. 2022;50(12):1809–18. 36094523 10.1097/CCM.0000000000005673

[28] Combes A, Hajage D, Capellier G, Demoule A, Lavoué S, Guervilly C, et al. Extracorporeal Membrane Oxygenation for Severe Acute Respiratory Distress Syndrome. N Engl J Med. 2018;378(21):1965–75. 29791822 10.1056/NEJMoa1800385

[29] Grasselli G, Calfee CS, Camporota L, Poole D, Amato MBP, Antonelli M, et al. ESICM guidelines on acute respiratory distress syndrome: definition, phenotyping and respiratory support strategies. Intensive Care Med. 2023;49(7):727–59. 37326646 10.1007/s00134-023-07050-7 PMC10354163

[30] Herridge MS, Chu LM, Matte A, Tomlinson G, Chan L, Thomas C, et al. The RECOVER Program: Disability Risk Groups and 1-Year Outcome after 7 or More Days of Mechanical Ventilation. Am J Respir Crit Care Med. 2016;194(7):831–44. 26974173 10.1164/rccm.201512-2343OC

[31] Herridge MS, Tansey CM, Matté A, Tomlinson G, Diaz-Granados N, Cooper A, et al. Functional Disability 5 Years after Acute Respiratory Distress Syndrome. N Engl J Med. 2011;364(14):1293–304. 21470008 10.1056/NEJMoa1011802

