# Supplementary material for: Complications in acute respiratory distress syndrome: a systematic review and meta-analysis
Source: Crit Care. 2026 Mar 29;30:238. doi: 10.1186/s13054-026-05978-y (PMC13151369; doi:10.1186/s13054-026-05978-y)

#
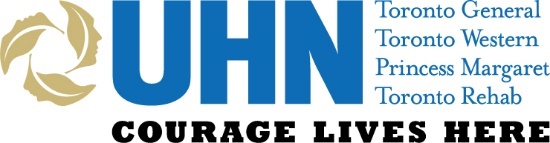
UHN Health Sciences Libraries

# Literature Search Results

**#201924; KS Sys Rev : update : ARDS + Mechanical Ventilation + Complications**

For: Drs E Fan & David Granton

Department: Critical Care

Date Completed: August 17, 2023

Tel:

Fax:

Email:

**Following is your update systematic review search for**:

**ARDS + Mechanical Ventilation + Complications or Etiology or Harm**; limited to English, human, adults. No conference abstracts. **Updated from June 1, 2019 to present**

**The databases searched were**:

1. [Medline](#Medline); 2. [Medline In-Process/ePubs](#Medline_In_Process); 3. [Embase](#Embase); 4. [CCTR](#CCTR); 5. [CDSR](#CDSR); 6. [ClinicalTrials.Gov](#ClinicalTrials_Gov)

**RESULTS & STRATEGY USED**: *see following*

**Search Completed By:** Marina Englesakis, Information Specialist

You may contact me either by telephone at (416) 340-4800 x3022 or via e-mail at [*marina.englesakis@uhn.ca*](mailto:marina.englesakis@uhn.ca)*.*

It is important that you are satisfied with your search results.

If you have any questions regarding this search, or if the results were not satisfactory, please do not hesitate in contacting me.

To request items not available in our library system, an INTERLIBRARY LOAN REQUEST FORM can be obtained from the library’s circulation desk or through the Virtual Library (<http://www.uhn.ca/Education/libraries/ill.asp> ). Any questions regarding our Document Delivery Service can be directed to Mellisha by telephone at 416-340-4121 or by email at [Mellisha.McKenzie@uhn.ca](mailto:Mellisha.McKenzie@uhn.ca).

Search strategy saved as 2023-08-17 EF - ARDS and Mechanical Ventilation or Artificial Respiration Injury and Etiology or Causation or Harm - UPDATE from 2019

Contents

[UHN Health Sciences Libraries 1](#_Toc143262069)

[Literature Search Results 1](#_Toc143262070)

[SEARCH HISTORY 2](#_Toc143262071)

[**Databases (Platforms)** 2](#_Toc143262072)

[Medline 3](#_Toc143262073)

[Medline In-Process 8](#_Toc143262074)

[Embase 14](#_Toc143262075)

[CCTR 21](#_Toc143262076)

[CDSR 26](#_Toc143262077)

[ClinicalTrials.Gov 29](#_Toc143262078)

# SEARCH HISTORY

| **Databases (Platforms)** | **Database Dates covered** | **Date Database  was searched** | **# Citations** | **Notes/Comments** |
| --- | --- | --- | --- | --- |
| MEDLINE (Ovid) | 1946 – Aug. 16, 2023 | Aug. 17, 2023 | 2522 |  |
| MEDLINE ePub Ahead of Print / MEDLINE In-Process & Other Non-Indexed Citations (Ovid) | 2023 Aug. 16 | Aug. 17, 2023 | 917 |  |
| Embase Classic+Embase (Ovid) | 1947 – Aug. 16, 2023 | Aug. 17, 2023 | 5965 | Conference, non-journal materials removed at source |
| Cochrane Central Register of Controlled Trials (Ovid) | 1991 – July 2023 | Aug. 17, 2023 | 271 | Conference, non-journal materials removed at source |
| Cochrane Database of Systematic Reviews (Ovid) | 2005 – Aug. 16, 2023 | Aug. 17, 2023 | 4 |  |
|  |  | Totals: | 9679 | Results in RIS File(s) |
| **Clinical Trial Registries** |  |  |  |  |
| ClinicalTrials.Gov | n/a | Aug. 17, 2023 | 18 |  |
|  |  |  |  |  |

# Medline

Ovid MEDLINE(R) 1946 to August 16, 2023

| **#** | **Searches** | **Results** |
| --- | --- | --- |
| 1 | Respiratory Distress Syndrome/ [MeSH 2021; see RESPIRATORY DISTRESS SYNDROME, ADULT 1977-2020 ] | 24779 |
| 2 | Respiratory Distress Syndrome, Adult/ | 24779 |
| 3 | acute respiratory distress syndrome?.mp. | 17926 |
| 4 | adult respiratory distress syndrome?.mp. | 4243 |
| 5 | APRONet.mp. | 1 |
| 6 | ARDS.mp. | 15739 |
| 7 | ARDSnet*.mp. | 121 |
| 8 | ARDSS.mp. | 8 |
| 9 | shock lung.mp. | 417 |
| 10 | Acute Chest Syndrome/ [MeSH as of 2010; related to ARDS] | 384 |
| 11 | Acute Lung Injury/ | 8513 |
| 12 | (acute adj1 chest adj1 syndrome?).mp. | 1198 |
| 13 | (acute adj1 lung? adj1 fail*).mp. | 138 |
| 14 | (acute adj1 pulmonary adj1 fail*).mp. | 105 |
| 15 | (acute adj1 bronchopulmonary adj1 fail*).mp. | 0 |
| 16 | (acute adj1 respirat* adj1 fail*).mp. | 7459 |
| 17 | (acute adj1 lung? adj1 injur*).mp. | 16916 |
| 18 | (acute adj1 pulmonary adj1 injur*).mp. | 141 |
| 19 | (acute adj1 bronchopulmonary adj1 injur*).mp. | 0 |
| 20 | (acute adj1 respirat* adj1 injur*).mp. | 514 |
| 21 | ALI.ti,ab. [ Acute Lung Injury ] | 8326 |
| 22 | or/1-21 [ ARDS & related terms ] | 56150 |
| 23 | exp ventilators, mechanical/ | 10251 |
| 24 | ventilators, negative-pressure/ | 402 |
| 25 | exp respiratory therapy/ | 133602 |
| 26 | exp respiration, artificial/ | 89136 |
| 27 | (respirator or respirators).mp. | 6556 |
| 28 | (ventilator or ventilators).mp. | 38381 |
| 29 | (mechan* adj5 ventilat*).mp. | 69162 |
| 30 | (pressure? adj5 ventilat*).mp. | 17479 |
| 31 | (respirator* adj2 therap*).mp. | 10633 |
| 32 | (inhalat* adj2 therap*).mp. | 18231 |
| 33 | (oxygen* adj2 therap*).mp. | 25715 |
| 34 | ventilation.mp. | 148899 |
| 35 | ventilatory.mp. | 30955 |
| 36 | (art#ficial adj1 respiration).mp. | 57557 |
| 37 | (art#ficial adj1 breath*).mp. | 58 |
| 38 | (ventilat* adj2 support*).mp. | 9823 |
| 39 | (respirator* adj2 support*).mp. | 6285 |
| 40 | (breath* adj3 support*).mp. | 562 |
| 41 | continuous positive airway pressure?.mp. | 13844 |
| 42 | (hyperbaric adj oxygenat*).mp. | 12872 |
| 43 | (intermittent* adj1 positive-pressure?).mp. | 4154 |
| 44 | (positive-pressure adj1 respirat*).mp. | 18656 |
| 45 | cpap.mp. | 9085 |
| 46 | peep.mp. | 5688 |
| 47 | wean*3.mp. | 48757 |
| 48 | or/25-47 [ Mechanical Ventilation & related terms including "Artificial Respiration" ] | 317338 |
| 49 | 22 and 48 [ ARDS + Mechanical Ventilation ] | 20289 |
| 50 | exp risk/ | 1385173 |
| 51 | risk*3.mp. | 2964662 |
| 52 | cohort?.mp. | 851512 |
| 53 | (cause? or causing or causal* or causation).mp. | 2665978 |
| 54 | exp odds ratio/ | 97820 |
| 55 | et.fs. [ Etiology floating subheading ] | 2775924 |
| 56 | (harm or harms or harmed or harming or harmful*).mp. | 141762 |
| 57 | aetiolog*.mp. | 66972 |
| 58 | etiolog*.mp. | 2952751 |
| 59 | "hazard analysis and critical control points"/ or patient harm/ | 516 |
| 60 | Iatrogenic Disease/ | 18119 |
| 61 | ae.fs. [ Adverse Effects floating subheaading ] | 1999164 |
| 62 | (adverse?? adj2 effect?).mp. | 2150271 |
| 63 | (adverse?? adj2 event?).mp. | 215470 |
| 64 | in.fs. [ Injuries floating subheading ] | 247939 |
| 65 | mo.fs. [ Mortality floating subheading ] | 628570 |
| 66 | exp Mortality/ | 423938 |
| 67 | exp Morbidity/ | 650522 |
| 68 | exp Prognosis/ | 1924890 |
| 69 | Survival/ | 4924 |
| 70 | exp Survival Analysis/ | 334002 |
| 71 | Survival Rate/ | 189856 |
| 72 | Survivors/ | 30533 |
| 73 | surviv*.mp. | 1549050 |
| 74 | exp Treatment Outcome/ | 1248797 |
| 75 | co.fs. [ Complications floating subheading ] | 2232554 |
| 76 | complication?.mp. | 3351374 |
| 77 | outcome?.mp. | 2828122 |
| 78 | exp health status indicators/ | 342763 |
| 79 | exp Probability/ | 1602512 |
| 80 | odds ratio*.mp. | 363646 |
| 81 | iatrogen*.mp. | 40864 |
| 82 | death??.mp. | 997347 |
| 83 | morbidit*.mp. | 425135 |
| 84 | mortalit*.mp. | 1268758 |
| 85 | exp Barotrauma/ | 9861 |
| 86 | exp Ventilator-Induced Lung Injury/ | 7331 |
| 87 | Bronchopulmonary Dysplasia/ | 6072 |
| 88 | exp Pneumothorax, Artificial/ | 2177 |
| 89 | exp Pneumothorax/ | 18397 |
| 90 | exp Mediastinal Emphysema/ | 3985 |
| 91 | exp Pneumopericardium/ | 978 |
| 92 | exp Subcutaneous Emphysema/ | 6418 |
| 93 | exp Tracheal Diseases/ | 18971 |
| 94 | exp stomach ulcer/ | 26950 |
| 95 | exp peptic ulcer perforation/ | 6018 |
| 96 | exp Gastrointestinal Hemorrhage/ | 54367 |
| 97 | exp Arrhythmias, Cardiac/ | 238159 |
| 98 | exp Myocardial Infarction/ | 193970 |
| 99 | exp cerebrovascular disorders/ | 429195 |
| 100 | exp Pneumonia, Ventilator-Associated/ | 4280 |
| 101 | exp Pulmonary Atelectasis/ | 7057 |
| 102 | Muscle Weakness/ | 9654 |
| 103 | Diaphragm/ | 21924 |
| 104 | exp thromboembolism/ | 64402 |
| 105 | exp venous thromboembolism/ | 15797 |
| 106 | exp Hemorrhage/ | 364839 |
| 107 | exp "Quality of Life"/ | 271010 |
| 108 | arrhythmia?.mp. | 145662 |
| 109 | atelectas#s.mp. | 11365 |
| 110 | barotrauma*.mp. | 3253 |
| 111 | bleed*.mp. | 218338 |
| 112 | bronchopulmonary dysplasia*.mp. | 9208 |
| 113 | CVA.ti,ab. | 2845 |
| 114 | deep venous thrombos#s.mp. | 11809 |
| 115 | diaphragm?.mp. | 38634 |
| 116 | DVT.ti,ab. | 10584 |
| 117 | h?emorrhage?.mp. | 329771 |
| 118 | HRQoL.mp. | 19702 |
| 119 | (lung? adj3 collaps*).mp. | 1761 |
| 120 | (lung? adj4 injur*).mp. | 41077 |
| 121 | morbidity.mp. | 404286 |
| 122 | mortalit*3.mp. | 1268638 |
| 123 | myocardial infarction?.mp. | 257650 |
| 124 | PE.ti,ab. | 54366 |
| 125 | Pneumomediastinum.mp. | 3619 |
| 126 | pneumonia?.mp. | 263706 |
| 127 | pneumopericardium.mp. | 1116 |
| 128 | pneumothoraces.mp. | 1844 |
| 129 | pneumothorax.mp. | 27347 |
| 130 | pulmonary emboli*.mp. | 57103 |
| 131 | (pulmonary adj4 injur*).mp. | 6211 |
| 132 | QALI.ti,ab. | 1 |
| 133 | QALY.ti,ab. | 10059 |
| 134 | QoL.ti,ab. | 43655 |
| 135 | shock.mp. | 233468 |
| 136 | troponin*.mp. | 32090 |
| 137 | ulcer?.mp. | 185443 |
| 138 | VAP.ti,ab. | 4342 |
| 139 | VTE.ti,ab. | 12551 |
| 140 | (stroke or strokes).mp. | 334607 |
| 141 | subcutaneous emphysema.mp. | 3981 |
| 142 | (tracheal adj1 injur*).mp. | 569 |
| 143 | (tracheal adj1 stenos#s).mp. | 6834 |
| 144 | (ventilator adj2 lung adj2 injur*).mp. | 2369 |
| 145 | exp Lung Diseases/ and exp Iatrogenic Disease/ | 11698 |
| 146 | exp Lungs/ and exp Iatrogenic Disease/ | 602 |
| 147 | exp Lung Injury/ and exp Iatrogenic Disease/ | 158 |
| 148 | (lung? adj4 iatrogen*).mp. | 90 |
| 149 | (pulmonary adj4 iatrogen*).mp. | 166 |
| 150 | or/50-149 [ Etiology / Causation / Harm / Named Injuries ] | 13107775 |
| 151 | 49 and 150 [ ARDS + Mechanical Ventilation + Harm ] | 17774 |
| 152 | Randomized controlled trial.pt. | 597766 |
| 153 | exp Randomized controlled trial/ | 599306 |
| 154 | exp Randomized Controlled Trials as Topic/ | 167319 |
| 155 | Pragmatic Clinical Trial.pt. | 2240 |
| 156 | Pragmatic Clinical Trial/ | 2240 |
| 157 | Pragmatic Clinical Trials As Topic/ | 867 |
| 158 | (pragmatic adj2 (trial? or study or studies)).mp,kw. | 5888 |
| 159 | random*.mp,kw. | 1463766 |
| 160 | Double-Blind Method/ | 175924 |
| 161 | ((single or double or triple or treble) adj3 (blind* or mask*)).mp,kw. | 252383 |
| 162 | doubleblind*.mp,kw. | 216 |
| 163 | Placebos/ | 35932 |
| 164 | Placebo*.mp,kw. | 240755 |
| 165 | cohort*.mp,kw. | 852111 |
| 166 | evaluation studies.pt. | 0 |
| 167 | (evaluation adj1 (study or studies)).mp,kw. | 390677 |
| 168 | validation studies.pt. | 0 |
| 169 | (validation adj1 (study or studies)).mp,kw. | 124734 |
| 170 | clinical trial.pt. | 537786 |
| 171 | Controlled Clinical Trial.pt. | 95382 |
| 172 | Controlled Clinical Trial/ | 95382 |
| 173 | Controlled Clinical Trials As Topic/ | 5657 |
| 174 | (controlled adj1 clinical adj2 (trial? or study or studies)).mp,kw. | 132566 |
| 175 | meta-analysis/ | 185415 |
| 176 | meta-analysis as topic/ | 22513 |
| 177 | (meta-anal* or metanal* or metaanal*).mp,kw. | 262594 |
| 178 | (systematic adj4 (review or reviews or overview or overviews)).mp,kw. | 275486 |
| 179 | (overview? adj4 (review or reviews)).mp,kw. | 22440 |
| 180 | exp Cohort Studies/ | 2510438 |
| 181 | exp Case-Control Studies/ | 1436922 |
| 182 | (case control* adj2 (study or studies)).mp,kw. | 356234 |
| 183 | Longitudinal Studies/ | 166430 |
| 184 | (longitudinal* adj2 (study or studies)).mp,kw. | 208638 |
| 185 | Prospective Studies/ | 665380 |
| 186 | (prospective* adj2 (study or studies)).mp,kw. | 761685 |
| 187 | Cross-Sectional Studies/ | 474561 |
| 188 | (cross-sectional* adj2 (study or studies)).mp,kw. | 499967 |
| 189 | Retrospective Studies/ | 1137629 |
| 190 | (retrospective* adj2 (study or studies)).mp,kw. | 1174757 |
| 191 | Comparative Study.pt. | 1912901 |
| 192 | Comparative Study/ | 1912901 |
| 193 | (comparative adj2 (trial? or study or studies)).mp,kw. | 1977362 |
| 194 | Multicenter Study.pt. | 336762 |
| 195 | Multicenter Study/ | 336762 |
| 196 | ((multicenter* or multicentre*) adj2 (trial? or study or studies)).mp,kw. | 377843 |
| 197 | Multicenter Studies as Topic/ | 22696 |
| 198 | equivalence trial/ or "equivalence trial (topic)"/ | 1139 |
| 199 | (conceal* adj2 allocat*).mp,kw. | 3130 |
| 200 | (equivalen* adj4 (trial? or study or studies)).mp,kw. | 6278 |
| 201 | (superiority adj4 (trial? or study or studies)).mp,kw. | 4231 |
| 202 | ((noninferiority or non-inferiority) adj4 (trial? or study or studies)).mp,kw. | 6341 |
| 203 | Clinical Trial, Phase III/ | 21925 |
| 204 | Clinical Trial, Phase III.pt. | 21925 |
| 205 | Clinical Trials, Phase III as Topic/ | 11129 |
| 206 | ("phase 3" or "phase3" or "phase III").mp. | 65694 |
| 207 | or/152-206 [ Studies ] | 6798147 |
| 208 | 151 and 207 [ ARDS + Mechanical Ventilation + Harm + Studies ] | 8632 |
| 209 | limit 208 to english language | 8040 |
| 210 | exp animals/ not (exp animals/ and exp humans/) | 5146438 |
| 211 | 209 not 210 | 7153 |
| 212 | limit 209 to humans | 7146 |
| 213 | 211 or 212 | 7153 |
| 214 | limit 213 to ("all infant (birth to 23 months)" or "all child (0 to 18 years)" or "newborn infant (birth to 1 month)" or "infant (1 to 23 months)" or "preschool child (2 to 5 years)" or "child (6 to 12 years)" or "adolescent (13 to 18 years)") | 1390 |
| 215 | 213 not 214 | 5763 |
| 216 | limit 213 to ("all adult (19 plus years)" or "young adult (19 to 24 years)" or "adult (19 to 44 years)" or "young adult and adult (19-24 and 19-44)" or "middle age (45 to 64 years)" or "middle aged (45 plus years)" or "all aged (65 and over)" or "aged (80 and over)") | 4655 |
| 217 | 215 or 216 | 6414 |
| 218 | 20190601:20241231.(da). | 4816865 |
| 219 | 20190601:20241231.(dt). | 4070395 |
| 220 | 20190601:20241231.(ep). | 3180570 |
| 221 | 20190601:20241231.(ez). | 4066907 |
| 222 | 218 or 219 or 220 or 221 | 4817051 |
| 223 | 217 and 222 [ ARDS + Mechanical Ventilation + Harm + Studies; limits applied. Update Period Only ] | 2554 |
| 224 | remove duplicates from 223 [ removal of internal database duplicats ] | 2522 |
| 225 | 224 [ FINAL: ARDS + Mechanical Ventilation + Harm + Studies; limits applied. Update Period Only ] | 2522 |

# Medline In-Process

Ovid MEDLINE(R) Epub Ahead of Print and In-Process, In-Data-Review & Other Non-Indexed Citations August 16, 2023

| **#** | **Searches** | **Results** |
| --- | --- | --- |
| 1 | Respiratory Distress Syndrome/ [MeSH 2021; see RESPIRATORY DISTRESS SYNDROME, ADULT 1977-2020 ] | 0 |
| 2 | Respiratory Distress Syndrome, Adult/ | 0 |
| 3 | acute respiratory distress syndrome?.mp. | 5090 |
| 4 | adult respiratory distress syndrome?.mp. | 157 |
| 5 | APRONet.mp. | 0 |
| 6 | ARDS.mp. | 3936 |
| 7 | ARDSnet*.mp. | 19 |
| 8 | ARDSS.mp. | 3 |
| 9 | shock lung.mp. | 4 |
| 10 | Acute Chest Syndrome/ [MeSH as of 2010; related to ARDS] | 0 |
| 11 | Acute Lung Injury/ | 0 |
| 12 | (acute adj1 chest adj1 syndrome?).mp. | 217 |
| 13 | (acute adj1 lung? adj1 fail*).mp. | 13 |
| 14 | (acute adj1 pulmonary adj1 fail*).mp. | 6 |
| 15 | (acute adj1 bronchopulmonary adj1 fail*).mp. | 0 |
| 16 | (acute adj1 respirat* adj1 fail*).mp. | 1477 |
| 17 | (acute adj1 lung? adj1 injur*).mp. | 1965 |
| 18 | (acute adj1 pulmonary adj1 injur*).mp. | 10 |
| 19 | (acute adj1 bronchopulmonary adj1 injur*).mp. | 0 |
| 20 | (acute adj1 respirat* adj1 injur*).mp. | 84 |
| 21 | ALI.ti,ab. [ Acute Lung Injury ] | 1985 |
| 22 | or/1-21 [ ARDS & related terms ] | 9970 |
| 23 | exp ventilators, mechanical/ | 0 |
| 24 | ventilators, negative-pressure/ | 0 |
| 25 | exp respiratory therapy/ | 0 |
| 26 | exp respiration, artificial/ | 0 |
| 27 | (respirator or respirators).mp. | 640 |
| 28 | (ventilator or ventilators).mp. | 5026 |
| 29 | (mechan* adj5 ventilat*).mp. | 11210 |
| 30 | (pressure? adj5 ventilat*).mp. | 1945 |
| 31 | (respirator* adj2 therap*).mp. | 659 |
| 32 | (inhalat* adj2 therap*).mp. | 307 |
| 33 | (oxygen* adj2 therap*).mp. | 2467 |
| 34 | ventilation.mp. | 19412 |
| 35 | ventilatory.mp. | 2692 |
| 36 | (art#ficial adj1 respiration).mp. | 201 |
| 37 | (art#ficial adj1 breath*).mp. | 3 |
| 38 | (ventilat* adj2 support*).mp. | 1564 |
| 39 | (respirator* adj2 support*).mp. | 1337 |
| 40 | (breath* adj3 support*).mp. | 105 |
| 41 | continuous positive airway pressure?.mp. | 1577 |
| 42 | (hyperbaric adj oxygenat*).mp. | 93 |
| 43 | (intermittent* adj1 positive-pressure?).mp. | 108 |
| 44 | (positive-pressure adj1 respirat*).mp. | 31 |
| 45 | cpap.mp. | 1473 |
| 46 | peep.mp. | 754 |
| 47 | wean*3.mp. | 7106 |
| 48 | or/25-47 [ Mechanical Ventilation & related terms including "Artificial Respiration" ] | 35343 |
| 49 | 22 and 48 [ ARDS + Mechanical Ventilation ] | 3291 |
| 50 | exp risk/ | 14 |
| 51 | risk*3.mp. | 411162 |
| 52 | cohort?.mp. | 125338 |
| 53 | (cause? or causing or causal* or causation).mp. | 470188 |
| 54 | exp odds ratio/ | 0 |
| 55 | et.fs. [ Etiology floating subheading ] | 0 |
| 56 | (harm or harms or harmed or harming or harmful*).mp. | 28601 |
| 57 | aetiolog*.mp. | 7227 |
| 58 | etiolog*.mp. | 46075 |
| 59 | "hazard analysis and critical control points"/ or patient harm/ | 0 |
| 60 | Iatrogenic Disease/ | 0 |
| 61 | ae.fs. [ Adverse Effects floating subheaading ] | 5 |
| 62 | (adverse?? adj2 effect?).mp. | 33903 |
| 63 | (adverse?? adj2 event?).mp. | 41081 |
| 64 | in.fs. [ Injuries floating subheading ] | 0 |
| 65 | mo.fs. [ Mortality floating subheading ] | 0 |
| 66 | exp Mortality/ | 0 |
| 67 | exp Morbidity/ | 3 |
| 68 | exp Prognosis/ | 1 |
| 69 | Survival/ | 0 |
| 70 | exp Survival Analysis/ | 0 |
| 71 | Survival Rate/ | 0 |
| 72 | Survivors/ | 0 |
| 73 | surviv*.mp. | 191158 |
| 74 | exp Treatment Outcome/ | 0 |
| 75 | co.fs. [ Complications floating subheading ] | 0 |
| 76 | complication?.mp. | 171056 |
| 77 | outcome?.mp. | 362088 |
| 78 | exp health status indicators/ | 0 |
| 79 | exp Probability/ | 155 |
| 80 | odds ratio*.mp. | 44658 |
| 81 | iatrogen*.mp. | 6332 |
| 82 | death??.mp. | 127005 |
| 83 | morbidit*.mp. | 69950 |
| 84 | mortalit*.mp. | 142374 |
| 85 | exp Barotrauma/ | 0 |
| 86 | exp Ventilator-Induced Lung Injury/ | 0 |
| 87 | Bronchopulmonary Dysplasia/ | 0 |
| 88 | exp Pneumothorax, Artificial/ | 0 |
| 89 | exp Pneumothorax/ | 0 |
| 90 | exp Mediastinal Emphysema/ | 0 |
| 91 | exp Pneumopericardium/ | 0 |
| 92 | exp Subcutaneous Emphysema/ | 0 |
| 93 | exp Tracheal Diseases/ | 0 |
| 94 | exp stomach ulcer/ | 0 |
| 95 | exp peptic ulcer perforation/ | 0 |
| 96 | exp Gastrointestinal Hemorrhage/ | 0 |
| 97 | exp Arrhythmias, Cardiac/ | 1 |
| 98 | exp Myocardial Infarction/ | 0 |
| 99 | exp cerebrovascular disorders/ | 0 |
| 100 | exp Pneumonia, Ventilator-Associated/ | 0 |
| 101 | exp Pulmonary Atelectasis/ | 0 |
| 102 | Muscle Weakness/ | 0 |
| 103 | Diaphragm/ | 0 |
| 104 | exp thromboembolism/ | 0 |
| 105 | exp venous thromboembolism/ | 0 |
| 106 | exp Hemorrhage/ | 0 |
| 107 | exp "Quality of Life"/ | 5 |
| 108 | arrhythmia?.mp. | 12408 |
| 109 | atelectas#s.mp. | 1038 |
| 110 | barotrauma*.mp. | 345 |
| 111 | bleed*.mp. | 35719 |
| 112 | bronchopulmonary dysplasia*.mp. | 1033 |
| 113 | CVA.ti,ab. | 581 |
| 114 | deep venous thrombos#s.mp. | 1612 |
| 115 | diaphragm?.mp. | 3725 |
| 116 | DVT.ti,ab. | 2021 |
| 117 | h?emorrhage?.mp. | 28616 |
| 118 | HRQoL.mp. | 3341 |
| 119 | (lung? adj3 collaps*).mp. | 316 |
| 120 | (lung? adj4 injur*).mp. | 4875 |
| 121 | morbidity.mp. | 64968 |
| 122 | mortalit*3.mp. | 142330 |
| 123 | myocardial infarction?.mp. | 21281 |
| 124 | PE.ti,ab. | 9842 |
| 125 | Pneumomediastinum.mp. | 828 |
| 126 | pneumonia?.mp. | 30121 |
| 127 | pneumopericardium.mp. | 177 |
| 128 | pneumothoraces.mp. | 265 |
| 129 | pneumothorax.mp. | 3851 |
| 130 | pulmonary emboli*.mp. | 6421 |
| 131 | (pulmonary adj4 injur*).mp. | 757 |
| 132 | QALI.ti,ab. | 0 |
| 133 | QALY.ti,ab. | 1688 |
| 134 | QoL.ti,ab. | 8676 |
| 135 | shock.mp. | 27152 |
| 136 | troponin*.mp. | 4552 |
| 137 | ulcer?.mp. | 14970 |
| 138 | VAP.ti,ab. | 841 |
| 139 | VTE.ti,ab. | 2565 |
| 140 | (stroke or strokes).mp. | 46469 |
| 141 | subcutaneous emphysema.mp. | 753 |
| 142 | (tracheal adj1 injur*).mp. | 113 |
| 143 | (tracheal adj1 stenos#s).mp. | 445 |
| 144 | (ventilator adj2 lung adj2 injur*).mp. | 377 |
| 145 | exp Lung Diseases/ and exp Iatrogenic Disease/ | 0 |
| 146 | exp Lungs/ and exp Iatrogenic Disease/ | 0 |
| 147 | exp Lung Injury/ and exp Iatrogenic Disease/ | 0 |
| 148 | (lung? adj4 iatrogen*).mp. | 17 |
| 149 | (pulmonary adj4 iatrogen*).mp. | 36 |
| 150 | or/50-149 [ Etiology / Causation / Harm / Named Injuries ] | 1474978 |
| 151 | 49 and 150 [ ARDS + Mechanical Ventilation + Harm ] | 2928 |
| 152 | Randomized controlled trial.pt. | 288 |
| 153 | exp Randomized controlled trial/ | 288 |
| 154 | exp Randomized Controlled Trials as Topic/ | 0 |
| 155 | Pragmatic Clinical Trial.pt. | 0 |
| 156 | Pragmatic Clinical Trial/ | 0 |
| 157 | Pragmatic Clinical Trials As Topic/ | 0 |
| 158 | (pragmatic adj2 (trial? or study or studies)).mp,kw. | 661 |
| 159 | random*.mp,kw. | 224257 |
| 160 | Double-Blind Method/ | 0 |
| 161 | ((single or double or triple or treble) adj3 (blind* or mask*)).mp,kw. | 18912 |
| 162 | doubleblind*.mp,kw. | 26 |
| 163 | Placebos/ | 0 |
| 164 | Placebo*.mp,kw. | 22231 |
| 165 | cohort*.mp,kw. | 125414 |
| 166 | evaluation studies.pt. | 26 |
| 167 | (evaluation adj1 (study or studies)).mp,kw. | 1385 |
| 168 | validation studies.pt. | 0 |
| 169 | (validation adj1 (study or studies)).mp,kw. | 3813 |
| 170 | clinical trial.pt. | 784 |
| 171 | Controlled Clinical Trial.pt. | 21 |
| 172 | Controlled Clinical Trial/ | 21 |
| 173 | Controlled Clinical Trials As Topic/ | 0 |
| 174 | (controlled adj1 clinical adj2 (trial? or study or studies)).mp,kw. | 5257 |
| 175 | meta-analysis/ | 120 |
| 176 | meta-analysis as topic/ | 0 |
| 177 | (meta-anal* or metanal* or metaanal*).mp,kw. | 49675 |
| 178 | (systematic adj4 (review or reviews or overview or overviews)).mp,kw. | 65026 |
| 179 | (overview? adj4 (review or reviews)).mp,kw. | 7080 |
| 180 | exp Cohort Studies/ | 4 |
| 181 | exp Case-Control Studies/ | 2 |
| 182 | (case control* adj2 (study or studies)).mp,kw. | 17644 |
| 183 | Longitudinal Studies/ | 0 |
| 184 | (longitudinal* adj2 (study or studies)).mp,kw. | 16145 |
| 185 | Prospective Studies/ | 2 |
| 186 | (prospective* adj2 (study or studies)).mp,kw. | 47836 |
| 187 | Cross-Sectional Studies/ | 4 |
| 188 | (cross-sectional* adj2 (study or studies)).mp,kw. | 65078 |
| 189 | Retrospective Studies/ | 2 |
| 190 | (retrospective* adj2 (study or studies)).mp,kw. | 66847 |
| 191 | Comparative Study.pt. | 47 |
| 192 | Comparative Study/ | 47 |
| 193 | (comparative adj2 (trial? or study or studies)).mp,kw. | 22497 |
| 194 | Multicenter Study.pt. | 10 |
| 195 | Multicenter Study/ | 10 |
| 196 | ((multicenter* or multicentre*) adj2 (trial? or study or studies)).mp,kw. | 11672 |
| 197 | Multicenter Studies as Topic/ | 0 |
| 198 | equivalence trial/ or "equivalence trial (topic)"/ | 0 |
| 199 | (conceal* adj2 allocat*).mp,kw. | 331 |
| 200 | (equivalen* adj4 (trial? or study or studies)).mp,kw. | 718 |
| 201 | (superiority adj4 (trial? or study or studies)).mp,kw. | 728 |
| 202 | ((noninferiority or non-inferiority) adj4 (trial? or study or studies)).mp,kw. | 913 |
| 203 | Clinical Trial, Phase III/ | 1 |
| 204 | Clinical Trial, Phase III.pt. | 1 |
| 205 | Clinical Trials, Phase III as Topic/ | 0 |
| 206 | ("phase 3" or "phase3" or "phase III").mp. | 8034 |
| 207 | or/152-206 [ Studies ] | 576469 |
| 208 | 151 and 207 [ ARDS + Mechanical Ventilation + Harm + Studies ] | 1210 |
| 209 | limit 208 to english language | 1196 |
| 210 | 20190601:20241231.(da). | 2120478 |
| 211 | 20190601:20241231.(dt). | 2260799 |
| 212 | 20190601:20241231.(ep). | 1852709 |
| 213 | 20190601:20241231.(ez). | 2120364 |
| 214 | 210 or 211 or 212 or 213 [ Update Period ] | 2261982 |
| 215 | 209 and 214 [ results limited to update period ] | 917 |
| 216 | remove duplicates from 215 [ removal of internal database duplicates ] | 917 |
| 217 | 216 [ FINAL: ARDS + Mechanical Ventilation + Harm + Studies; limits applied. Update Period Only ] | 917 |

# Embase

Embase Classic+Embase 1947 to 2023 August 16

| **#** | **Searches** | **Results** |
| --- | --- | --- |
| 1 | Respiratory Distress Syndrome/ [MeSH 2021; see RESPIRATORY DISTRESS SYNDROME, ADULT 1977-2020 ] | 17368 |
| 2 | Respiratory Distress Syndrome, Adult/ | 37089 |
| 3 | adult respiratory distress syndrome/ [ Embase ] | 55334 |
| 4 | acute respiratory distress syndrome?.mp. | 32423 |
| 5 | adult respiratory distress syndrome?.mp. | 57156 |
| 6 | APRONet.mp. | 4 |
| 7 | ARDS.mp. | 32238 |
| 8 | ARDSnet*.mp. | 431 |
| 9 | ARDSS.mp. | 14 |
| 10 | shock lung.mp. | 948 |
| 11 | Acute Chest Syndrome/ [MeSH as of 2010; related to ARDS] | 3202 |
| 12 | Acute Lung Injury/ | 18073 |
| 13 | (acute adj1 chest adj1 syndrome?).mp. | 3759 |
| 14 | (acute adj1 lung? adj1 fail*).mp. | 216 |
| 15 | (acute adj1 pulmonary adj1 fail*).mp. | 184 |
| 16 | (acute adj1 bronchopulmonary adj1 fail*).mp. | 0 |
| 17 | (acute adj1 respirat* adj1 fail*).mp. | 21972 |
| 18 | (acute adj1 lung? adj1 injur*).mp. | 30523 |
| 19 | (acute adj1 pulmonary adj1 injur*).mp. | 207 |
| 20 | (acute adj1 bronchopulmonary adj1 injur*).mp. | 0 |
| 21 | (acute adj1 respirat* adj1 injur*).mp. | 757 |
| 22 | ALI.ti,ab. [ Acute Lung Injury ] | 17407 |
| 23 | or/1-22 [ ARDS & related terms ] | 135816 |
| 24 | exp Ventilators, Mechanical/ | 6836 |
| 25 | Ventilators, Negative-Pressure/ | 53 |
| 26 | exp Respiratory Therapy/ | 5505 |
| 27 | exp Respiration, Artificial/ | 254286 |
| 28 | (respirator or respirators).mp. | 12084 |
| 29 | (ventilator or ventilators).mp. | 81209 |
| 30 | (mechan* adj5 ventilat*).mp. | 121823 |
| 31 | (pressure? adj5 ventilat*).mp. | 38573 |
| 32 | (respirator* adj2 therap*).mp. | 10047 |
| 33 | (inhalat* adj2 therap*).mp. | 3994 |
| 34 | (oxygen* adj2 therap*).mp. | 62185 |
| 35 | ventilation.mp. | 370010 |
| 36 | ventilatory.mp. | 50760 |
| 37 | (art#ficial adj1 respiration).mp. | 4355 |
| 38 | (art#ficial adj1 breath*).mp. | 104 |
| 39 | (ventilat* adj2 support*).mp. | 19765 |
| 40 | (respirator* adj2 support*).mp. | 12489 |
| 41 | (breath* adj3 support*).mp. | 1123 |
| 42 | continuous positive airway pressure?.mp. | 22418 |
| 43 | (hyperbaric adj oxygenat*).mp. | 3219 |
| 44 | (intermittent* adj1 positive-pressure?).mp. | 5791 |
| 45 | (positive-pressure adj1 respirat*).mp. | 993 |
| 46 | cpap.mp. | 21453 |
| 47 | peep.mp. | 11311 |
| 48 | wean*3.mp. | 80093 |
| 49 | exp artificial ventilation/ [ Embase ] | 254286 |
| 50 | exp ventilator/ [ Embase ] | 44945 |
| 51 | lung assist device/ [ Embase ] | 203 |
| 52 | mechanical ventilator/ [ Embase ] | 6836 |
| 53 | oxygen therapy/ [ Embase ] | 45782 |
| 54 | ventilated patient/ [ Embase ] | 8168 |
| 55 | ventilatory effort recorder/ [ Embase ] | 10 |
| 56 | or/26-55 [ Mechanical Ventilation & related terms including "Artificial Respiration" ] | 592076 |
| 57 | 23 and 56 [ ARDS + Mechanical Ventilation ] | 51607 |
| 58 | "Hazard Analysis and Critical Control Points"/ | 8450 |
| 59 | exp Health Status Indicators/ | 41256 |
| 60 | exp Morbidity/ | 445403 |
| 61 | exp Mortality/ | 1436005 |
| 62 | exp Odds Ratio/ | 29157 |
| 63 | exp Probability/ | 148400 |
| 64 | exp Prognosis/ | 959826 |
| 65 | exp Risk/ | 3118234 |
| 66 | exp Treatment Outcome/ | 2273423 |
| 67 | Iatrogenic Disease/ | 31476 |
| 68 | Patient Harm/ | 2855 |
| 69 | ae.fs. [ Adverse Effects floating subheaading ] | 1441080 |
| 70 | et.fs. [ Etiology floating subheading ] | 2672870 |
| 71 | [in.fs. Injuries floating subheading ] | 0 |
| 72 | [mo.fs. Mortality floating subheading ] | 0 |
| 73 | co.fs. [ Complications floating subheading ] | 2006936 |
| 74 | (adverse?? adj2 effect?).mp. | 433143 |
| 75 | (adverse?? adj2 event?).mp. | 499919 |
| 76 | (cause? or causing or causal* or causation).mp. | 4362860 |
| 77 | (harm or harms or harmed or harming or harmful*).mp. | 224033 |
| 78 | cohort?.mp. | 1674615 |
| 79 | complication?.mp. | 3644733 |
| 80 | death??.mp. | 1804706 |
| 81 | aetiolog*.mp. | 128042 |
| 82 | etiolog*.mp. | 3345766 |
| 83 | iatrogen*.mp. | 73205 |
| 84 | morbidit*.mp. | 866403 |
| 85 | mortalit*.mp. | 1976475 |
| 86 | odds ratio*.mp. | 473184 |
| 87 | outcome?.mp. | 4449068 |
| 88 | risk*3.mp. | 5111281 |
| 89 | exp Arrhythmias, Cardiac/ | 638293 |
| 90 | exp Barotrauma/ | 21950 |
| 91 | Bronchopulmonary Dysplasia/ | 9617 |
| 92 | exp Cerebrovascular Disorders/ | 829400 |
| 93 | Diaphragm/ | 38659 |
| 94 | exp Gastrointestinal Hemorrhage/ | 141920 |
| 95 | exp Hemorrhage/ | 1224170 |
| 96 | exp Lung Diseases/ and exp Iatrogenic Disease/ | 165296 |
| 97 | exp Lung Injury/ and exp Iatrogenic Disease/ | 2156 |
| 98 | exp Lung/ and exp Iatrogenic Disease/ | 11420 |
| 99 | exp Mediastinal Emphysema/ | 9158 |
| 100 | Muscle Weakness/ | 58368 |
| 101 | exp Myocardial Infarction/ | 473328 |
| 102 | exp Peptic Ulcer Perforation/ | 6163 |
| 103 | exp Pneumonia, Ventilator-Associated/ | 13757 |
| 104 | exp Pneumopericardium/ | 1844 |
| 105 | exp Pneumothorax, Artificial/ | 3501 |
| 106 | exp Pneumothorax/ | 57992 |
| 107 | exp Pulmonary Atelectasis/ | 27910 |
| 108 | exp "Quality of Life"/ | 649943 |
| 109 | exp Stomach Ulcer/ | 50581 |
| 110 | exp Subcutaneous Emphysema/ | 8002 |
| 111 | exp thromboembolism/ | 688280 |
| 112 | exp Tracheal Diseases/ | 35415 |
| 113 | exp venous thromboembolism/ | 206089 |
| 114 | exp Ventilator-Induced Lung Injury/ | 3011 |
| 115 | "health status indicat*".mp. | 4421 |
| 116 | "karnofsky performance".mp. | 17967 |
| 117 | "severity of illness*".mp. | 35802 |
| 118 | acute physiology scor*3.mp. | 6252 |
| 119 | (inpatient? adj2 acuit*).mp. | 66 |
| 120 | (lung? adj3 collaps*).mp. | 5289 |
| 121 | (lung? adj4 iatrogen*).mp. | 191 |
| 122 | (lung? adj4 injur*).mp. | 85057 |
| 123 | (myocardia* adj2 infarc*).mp. | 371126 |
| 124 | (organ? adj1 dysfunction adj1 scor*3).mp. | 1988 |
| 125 | (organ? adj2 fail*).mp. | 88576 |
| 126 | (patient? adj2 acuit*).mp. | 6572 |
| 127 | (pulmonary adj4 iatrogen*).mp. | 296 |
| 128 | (pulmonary adj4 injur*).mp. | 9564 |
| 129 | sickness impact?.mp. | 3416 |
| 130 | (stroke or strokes).mp. | 577369 |
| 131 | (tracheal adj1 injur*).mp. | 970 |
| 132 | (tracheal adj1 stenos#s).mp. | 5224 |
| 133 | (ventilator adj2 lung adj2 injur*).mp. | 4791 |
| 134 | APACHE.mp. | 30435 |
| 135 | arrhythmia?.mp. | 261028 |
| 136 | atelectas#s.mp. | 31465 |
| 137 | barotrauma*.mp. | 6197 |
| 138 | bleed*.mp. | 697596 |
| 139 | bronchopulmonary dysplasia*.mp. | 13066 |
| 140 | CVA.ti,ab. | 8204 |
| 141 | deep venous thrombos#s.mp. | 20400 |
| 142 | diaphragm?.mp. | 83708 |
| 143 | DVT.ti,ab. | 25077 |
| 144 | h?emorrhage?.mp. | 588938 |
| 145 | HLQoL.ti,ab. | 5 |
| 146 | HL-QoL.ti,ab. | 7 |
| 147 | morbidit*.mp. | 866403 |
| 148 | mortalit*3.mp. | 1976024 |
| 149 | myocardial infarction?.mp. | 358317 |
| 150 | pneumomediastinum.mp. | 9946 |
| 151 | pneumonia?.mp. | 540924 |
| 152 | pneumopericardium.mp. | 1983 |
| 153 | pneumothoraces.mp. | 3477 |
| 154 | pneumothorax.mp. | 65059 |
| 155 | pulmonary emboli*.mp. | 77106 |
| 156 | QALI.ti,ab. | 6 |
| 157 | QALY.ti,ab. | 21662 |
| 158 | QoL.ti,ab. | 97677 |
| 159 | shock.mp. | 425220 |
| 160 | subcutaneous emphysema.mp. | 9089 |
| 161 | thromboemboli*.mp. | 178619 |
| 162 | troponin*.mp. | 91738 |
| 163 | ulcer?.mp. | 342608 |
| 164 | VAP.ti,ab. | 8551 |
| 165 | VTE.ti,ab. | 29443 |
| 166 | etiology/ [ Embase ] | 388628 |
| 167 | lung barotrauma/ | 473 |
| 168 | exp atelectasis/ | 27910 |
| 169 | lung embolism/ | 126353 |
| 170 | lung congestion/ | 7911 |
| 171 | lung edema/ | 55740 |
| 172 | lung embolism/ | 126353 |
| 173 | lung hemorrhage/ | 17448 |
| 174 | lung infarction/ | 2867 |
| 175 | lung infection/ | 32977 |
| 176 | lung infiltrate/ | 17110 |
| 177 | lung injury/ | 43386 |
| 178 | lung insufficiency/ | 6176 |
| 179 | lung toxicity/ | 17304 |
| 180 | exp cerebrovascular accident/ | 314983 |
| 181 | exp troponin/ | 85771 |
| 182 | ventilator associated pneumonia/ | 13642 |
| 183 | or/58-182 [ Etiology or Causation or Harm or Named Injuries ] | 19241413 |
| 184 | 57 and 183 [ ARDS + Mechanical Ventilation + Etiology or Causation or Harm or Named Injuries ] | 47210 |
| 185 | exp Randomized controlled trial/ | 782719 |
| 186 | exp Randomized Controlled Trials as Topic/ | 259467 |
| 187 | Pragmatic Clinical Trial/ | 2268 |
| 188 | Pragmatic Clinical Trials As Topic/ | 259347 |
| 189 | (pragmatic adj2 (trial? or study or studies)).mp. | 6872 |
| 190 | random*.mp. | 2243838 |
| 191 | Double-Blind Method/ | 187136 |
| 192 | ((single or double or triple or treble) adj3 (blind* or mask*)).mp. | 370694 |
| 193 | doubleblind*.mp. | 4039 |
| 194 | Placebos/ | 354674 |
| 195 | Placebo*.mp. | 533629 |
| 196 | cohort*.mp. | 1675910 |
| 197 | (evaluation adj1 (study or studies)).mp. | 73230 |
| 198 | (validation adj1 (study or studies)).mp. | 120570 |
| 199 | Controlled Clinical Trial/ | 471117 |
| 200 | Controlled Clinical Trials As Topic/ | 11982 |
| 201 | (controlled adj1 clinical adj2 (trial? or study or studies)).mp. | 520823 |
| 202 | meta-analysis/ | 288517 |
| 203 | meta-analysis as topic/ | 40095 |
| 204 | (meta-anal* or metanal* or metaanal*).mp. | 448951 |
| 205 | (systematic adj4 (review or reviews or overview or overviews)).mp. | 555604 |
| 206 | (overview? adj4 (review or reviews)).mp. | 30207 |
| 207 | exp Cohort Studies/ | 1034774 |
| 208 | exp Case-Control Studies/ | 224286 |
| 209 | (case control* adj2 (study or studies)).mp. | 275380 |
| 210 | Longitudinal Studies/ | 176042 |
| 211 | (longitudinal* adj2 (study or studies)).mp. | 260286 |
| 212 | Prospective Studies/ | 766395 |
| 213 | (prospective* adj2 (study or studies)).mp. | 1079599 |
| 214 | Cross-Sectional Studies/ | 444700 |
| 215 | (cross-sectional* adj2 (study or studies)).mp. | 650343 |
| 216 | Retrospective Studies/ | 1195019 |
| 217 | (retrospective* adj2 (study or studies)).mp. | 1585884 |
| 218 | observational study/ | 331877 |
| 219 | Observational Studies as Topic/ | 331877 |
| 220 | (observational adj2 (study or studies)).mp. | 434222 |
| 221 | Controlled Before-After Studies/ [ New MeSH as of 2015 ] | 255777 |
| 222 | (cba adj1 (design? or procedure? or study or studies)).mp. | 223 |
| 223 | (before-after adj2 (design? or procedure? or study or studies)).mp. | 3598 |
| 224 | (before adj1 after adj2 (study or studies)).mp. | 2916 |
| 225 | Interrupted Time Series Analysis/ [ New MeSH as of 2015 ] | 248047 |
| 226 | (interrupt* adj1 time adj1 series).mp. | 7202 |
| 227 | Cross-Over Studies/ | 67890 |
| 228 | crossover procedure/ [ Embase] | 75371 |
| 229 | (cross over adj2 (design? or procedure? or study or studies or trial?)).mp. | 27381 |
| 230 | (crossover adj2 (design? or procedure? or study or studies or trial?)).mp. | 94250 |
| 231 | (conceal* adj2 allocat*).mp. | 4465 |
| 232 | Equivalence Trial/ | 165 |
| 233 | Equivalence Trials as Topic/ | 216 |
| 234 | (equivalen* adj4 (trial? or study or studies)).mp. | 7993 |
| 235 | ((noninferiority or non-inferiority) adj4 (trial? or study or studies)).mp. | 11648 |
| 236 | (superiority adj4 (trial? or study or studies)).mp. | 7682 |
| 237 | ("phase 3" or "phase3" or "phase III" or "P3" or "PIII" or "phase three").ti,ab,kw. | 159578 |
| 238 | or/185-237 [ "Therapy" Studies block ] | 7476760 |
| 239 | 184 and 238 [ ARDS + Mechanical Ventilation + Etiology or Causation or Harm or Named Injuries + Studies ] | 20216 |
| 240 | limit 239 to english language | 19423 |
| 241 | 240 not (addresses or bibliography or biography or book or book article or book book or book note or "book review" or book series article or book series article in press or book series chapter or book series conference paper or book series letter or "book series review" or book series short survey or chapter or monograph or book* or (abstract or conference or conference abstract or "conference proceeding" or "conference review" or journal conference abstract or "journal conference review" or conferenc*)).pt. | 13954 |
| 242 | 241 not ((book* or conferen* or chapter* or preprint* or report* or series or trade* or volume* or works or editorial).pt. or (medrxiv or biorxiv).so.) | 13639 |
| 243 | (exp animals/ or exp animal experimentation/ or nonhuman/) not ((exp animals/ or exp animal experimentation/ or nonhuman/) and exp human/) | 8029899 |
| 244 | 242 not 243 | 12771 |
| 245 | limit 242 to human | 12676 |
| 246 | 244 or 245 | 12771 |
| 247 | limit 246 to (embryo <first trimester> or infant <to one year> or child <unspecified age> or preschool child <1 to 6 years> or school child <7 to 12 years> or adolescent <13 to 17 years>) | 1606 |
| 248 | 246 not 247 | 11165 |
| 249 | limit 246 to (adult <18 to 64 years> or aged <65+ years>) | 7821 |
| 250 | 248 or 249 | 11792 |
| 251 | 20190601:20241231.(dc). [ Update Period ] | 8354458 |
| 252 | 250 and 251 | 5988 |
| 253 | remove duplicates from 252 [ removal of internal database duplicates ] | 5965 |
| 254 | 253 [ FINAL: ARDS + Mechanical Ventilation + Etiology or Causation or Harm or Named Injuries + Studies; limits applied. Update period only. ] | 5965 |
|  | | |

# CCTR

EBM Reviews - Cochrane Central Register of Controlled Trials July 2023

| **#** | **Searches** | **Results** |
| --- | --- | --- |
| 1 | Respiratory Distress Syndrome/ [MeSH 2021; see RESPIRATORY DISTRESS SYNDROME, ADULT 1977-2020 ] | 1907 |
| 2 | Respiratory Distress Syndrome, Adult/ | 68 |
| 3 | adult respiratory distress syndrome/ [ Embase ] | 219 |
| 4 | acute respiratory distress syndrome?.mp. | 2332 |
| 5 | adult respiratory distress syndrome?.mp. | 1584 |
| 6 | APRONet.mp. | 1 |
| 7 | ARDS.mp. | 2628 |
| 8 | ARDSnet*.mp. | 99 |
| 9 | ARDSS.mp. | 0 |
| 10 | shock lung.mp. | 9 |
| 11 | Acute Chest Syndrome/ [MeSH as of 2010; related to ARDS] | 61 |
| 12 | Acute Lung Injury/ | 700 |
| 13 | (acute adj1 chest adj1 syndrome?).mp. | 225 |
| 14 | (acute adj1 lung? adj1 fail*).mp. | 15 |
| 15 | (acute adj1 pulmonary adj1 fail*).mp. | 11 |
| 16 | (acute adj1 bronchopulmonary adj1 fail*).mp. | 0 |
| 17 | (acute adj1 respirat* adj1 fail*).mp. | 1512 |
| 18 | (acute adj1 lung? adj1 injur*).mp. | 1450 |
| 19 | (acute adj1 pulmonary adj1 injur*).mp. | 3 |
| 20 | (acute adj1 bronchopulmonary adj1 injur*).mp. | 0 |
| 21 | (acute adj1 respirat* adj1 injur*).mp. | 66 |
| 22 | ALI.ti,ab. [ Acute Lung Injury ] | 851 |
| 23 | or/1-22 [ ARDS & related terms ] | 6905 |
| 24 | exp Ventilators, Mechanical/ | 365 |
| 25 | Ventilators, Negative-Pressure/ | 34 |
| 26 | exp Respiratory Therapy/ | 10389 |
| 27 | exp Respiration, Artificial/ | 8169 |
| 28 | (respirator or respirators).mp. | 502 |
| 29 | (ventilator or ventilators).mp. | 8388 |
| 30 | (mechan* adj5 ventilat*).mp. | 15649 |
| 31 | (pressure? adj5 ventilat*).mp. | 7407 |
| 32 | (respirator* adj2 therap*).mp. | 2928 |
| 33 | (inhalat* adj2 therap*).mp. | 2155 |
| 34 | (oxygen* adj2 therap*).mp. | 6410 |
| 35 | ventilation.mp. | 34529 |
| 36 | ventilatory.mp. | 6090 |
| 37 | (art#ficial adj1 respiration).mp. | 4490 |
| 38 | (art#ficial adj1 breath*).mp. | 26 |
| 39 | (ventilat* adj2 support*).mp. | 2625 |
| 40 | (respirator* adj2 support*).mp. | 1863 |
| 41 | (breath* adj3 support*).mp. | 299 |
| 42 | continuous positive airway pressure?.mp. | 4732 |
| 43 | (hyperbaric adj oxygenat*).mp. | 606 |
| 44 | (intermittent* adj1 positive-pressure?).mp. | 780 |
| 45 | (positive-pressure adj1 respirat*).mp. | 1854 |
| 46 | cpap.mp. | 5142 |
| 47 | peep.mp. | 2325 |
| 48 | wean*3.mp. | 4544 |
| 49 | exp artificial ventilation/ [ Embase ] | 0 |
| 50 | exp ventilator/ [ Embase ] | 365 |
| 51 | lung assist device/ [ Embase ] | 1 |
| 52 | mechanical ventilator/ [ Embase ] | 17 |
| 53 | oxygen therapy/ [ Embase ] | 269 |
| 54 | ventilated patient/ [ Embase ] | 129 |
| 55 | ventilatory effort recorder/ [ Embase ] | 0 |
| 56 | or/26-55 [ Mechanical Ventilation & related terms including "Artificial Respiration" ] | 54976 |
| 57 | 23 and 56 [ ARDS + Mechanical Ventilation ] | 4179 |
| 58 | "Hazard Analysis and Critical Control Points"/ | 0 |
| 59 | exp Health Status Indicators/ | 26903 |
| 60 | exp Morbidity/ | 25827 |
| 61 | exp Mortality/ | 21511 |
| 62 | exp Odds Ratio/ | 4141 |
| 63 | exp Probability/ | 68768 |
| 64 | exp Prognosis/ | 205561 |
| 65 | exp Risk/ | 58260 |
| 66 | exp Treatment Outcome/ | 184629 |
| 67 | Iatrogenic Disease/ | 150 |
| 68 | Patient Harm/ | 15 |
| 69 | ae.fs. [ Adverse Effects floating subheaading ] | 157445 |
| 70 | et.fs. [ Etiology floating subheading ] | 86416 |
| 71 | [in.fs. Injuries floating subheading ] | 0 |
| 72 | [mo.fs. Mortality floating subheading ] | 0 |
| 73 | co.fs. [ Complications floating subheading ] | 68585 |
| 74 | (adverse?? adj2 effect?).mp. | 194508 |
| 75 | (adverse?? adj2 event?).mp. | 145140 |
| 76 | (cause? or causing or causal* or causation).mp. | 146074 |
| 77 | (harm or harms or harmed or harming or harmful*).mp. | 12058 |
| 78 | cohort?.mp. | 74923 |
| 79 | complication?.mp. | 223486 |
| 80 | death??.mp. | 87052 |
| 81 | aetiolog*.mp. | 2184 |
| 82 | etiolog*.mp. | 102683 |
| 83 | iatrogen*.mp. | 1608 |
| 84 | morbidit*.mp. | 46989 |
| 85 | mortalit*.mp. | 110986 |
| 86 | odds ratio*.mp. | 28381 |
| 87 | outcome?.mp. | 756847 |
| 88 | risk*3.mp. | 290349 |
| 89 | exp Arrhythmias, Cardiac/ | 13725 |
| 90 | exp Barotrauma/ | 132 |
| 91 | Bronchopulmonary Dysplasia/ | 632 |
| 92 | exp Cerebrovascular Disorders/ | 22274 |
| 93 | Diaphragm/ | 406 |
| 94 | exp Gastrointestinal Hemorrhage/ | 2480 |
| 95 | exp Hemorrhage/ | 18953 |
| 96 | exp Lung Diseases/ and exp Iatrogenic Disease/ | 879 |
| 97 | exp Lung Injury/ and exp Iatrogenic Disease/ | 7 |
| 98 | exp Lung/ and exp Iatrogenic Disease/ | 13 |
| 99 | exp Mediastinal Emphysema/ | 6 |
| 100 | Muscle Weakness/ | 805 |
| 101 | exp Myocardial Infarction/ | 13842 |
| 102 | exp Peptic Ulcer Perforation/ | 75 |
| 103 | exp Pneumonia, Ventilator-Associated/ | 538 |
| 104 | exp Pneumopericardium/ | 0 |
| 105 | exp Pneumothorax, Artificial/ | 10 |
| 106 | exp Pneumothorax/ | 535 |
| 107 | exp Pulmonary Atelectasis/ | 438 |
| 108 | exp "Quality of Life"/ | 42883 |
| 109 | exp Stomach Ulcer/ | 1195 |
| 110 | exp Subcutaneous Emphysema/ | 125 |
| 111 | exp thromboembolism/ | 3440 |
| 112 | exp Tracheal Diseases/ | 133 |
| 113 | exp venous thromboembolism/ | 1269 |
| 114 | exp Ventilator-Induced Lung Injury/ | 713 |
| 115 | "health status indicat*".mp. | 1212 |
| 116 | "karnofsky performance".mp. | 2308 |
| 117 | "severity of illness*".mp. | 24710 |
| 118 | acute physiology scor*3.mp. | 347 |
| 119 | (inpatient? adj2 acuit*).mp. | 3 |
| 120 | (lung? adj3 collaps*).mp. | 363 |
| 121 | (lung? adj4 iatrogen*).mp. | 11 |
| 122 | (lung? adj4 injur*).mp. | 3199 |
| 123 | (myocardia* adj2 infarc*).mp. | 35393 |
| 124 | (organ? adj1 dysfunction adj1 scor*3).mp. | 277 |
| 125 | (organ? adj2 fail*).mp. | 4421 |
| 126 | (patient? adj2 acuit*).mp. | 621 |
| 127 | (pulmonary adj4 iatrogen*).mp. | 5 |
| 128 | (pulmonary adj4 injur*).mp. | 323 |
| 129 | sickness impact?.mp. | 857 |
| 130 | (stroke or strokes).mp. | 68526 |
| 131 | (tracheal adj1 injur*).mp. | 24 |
| 132 | (tracheal adj1 stenos#s).mp. | 91 |
| 133 | (ventilator adj2 lung adj2 injur*).mp. | 279 |
| 134 | APACHE.mp. | 2996 |
| 135 | arrhythmia?.mp. | 13592 |
| 136 | atelectas#s.mp. | 1875 |
| 137 | barotrauma*.mp. | 280 |
| 138 | bleed*.mp. | 55936 |
| 139 | bronchopulmonary dysplasia*.mp. | 1689 |
| 140 | CVA.ti,ab. | 664 |
| 141 | deep venous thrombos#s.mp. | 1762 |
| 142 | diaphragm?.mp. | 2828 |
| 143 | DVT.ti,ab. | 2675 |
| 144 | h?emorrhage?.mp. | 35531 |
| 145 | HLQoL.ti,ab. | 0 |
| 146 | HL-QoL.ti,ab. | 1 |
| 147 | morbidit*.mp. | 46989 |
| 148 | mortalit*3.mp. | 110793 |
| 149 | myocardial infarction?.mp. | 35163 |
| 150 | pneumomediastinum.mp. | 64 |
| 151 | pneumonia?.mp. | 22489 |
| 152 | pneumopericardium.mp. | 2 |
| 153 | pneumothoraces.mp. | 115 |
| 154 | pneumothorax.mp. | 2578 |
| 155 | pulmonary emboli*.mp. | 4388 |
| 156 | QALI.ti,ab. | 0 |
| 157 | QALY.ti,ab. | 3868 |
| 158 | QoL.ti,ab. | 23995 |
| 159 | shock.mp. | 13157 |
| 160 | subcutaneous emphysema.mp. | 169 |
| 161 | thromboemboli*.mp. | 11481 |
| 162 | troponin*.mp. | 5240 |
| 163 | ulcer?.mp. | 23623 |
| 164 | VAP.ti,ab. | 1081 |
| 165 | VTE.ti,ab. | 2580 |
| 166 | etiology/ [ Embase ] | 348 |
| 167 | lung barotrauma/ | 2 |
| 168 | exp atelectasis/ | 438 |
| 169 | lung embolism/ | 352 |
| 170 | lung congestion/ | 25 |
| 171 | lung edema/ | 104 |
| 172 | lung embolism/ | 352 |
| 173 | lung hemorrhage/ | 41 |
| 174 | lung infarction/ | 1 |
| 175 | lung infection/ | 149 |
| 176 | lung infiltrate/ | 22 |
| 177 | lung injury/ | 417 |
| 178 | lung insufficiency/ | 14 |
| 179 | lung toxicity/ | 19 |
| 180 | exp cerebrovascular accident/ | 14700 |
| 181 | exp troponin/ | 1283 |
| 182 | ventilator associated pneumonia/ | 71 |
| 183 | or/58-182 [ Etiology or Causation or Harm or Named Injuries ] | 1286888 |
| 184 | 57 and 183 [ ARDS + Mechanical Ventilation + Etiology or Causation or Harm or Named Injuries ] | 3802 |
| 185 | limit 184 to english language | 3656 |
| 186 | 185 not (adolescence or adolescent or adolescents or babies or baby or boy or boys or child or childhood or children or childrens or children's or fetus or fetal or foetus or foetal or girl or girls or infancy or infant or infants or neonatal or neonatally or neonate or neonates or newborn or newborns or paediatric or paediatrician or paediatricians or paediatrics or pediatric or pediatrician or pediatricians or pediatrics or preschool* or teen or teenage or teenagers or teens or toddler or toddlers or tween* or youth or youths).ti,jw. | 3274 |
| 187 | 185 and (elder* or senior? or aged or adult* or man or men or woman* or women or maternal or young people or young person?).ti,jw. | 145 |
| 188 | 186 or 187 | 3280 |
| 189 | 188 not (abstract or addresses or bibliography or biography or book or book article or book book or book note or "book review" or book series article or book series article in press or book series chapter or book series conference paper or book series letter or "book series review" or book series short survey or chapter or conference or conference abstract or conference abstract placebo controlled partly blinded crossover study in 12 sle patients or conference proceeding or "conference review" or journal conference abstract or "journal conference review" or monograph or conferenc* or book* or trial* or protocol*).pt. | 1595 |
| 190 | 189 and ("2021-06*" or "2021-07*" or "2021-08*" or "2021-09*" or "2021-10*" or "2021-1*" or "2021-12*" or "2022*" or "2023*").dl. | 276 |
| 191 | remove duplicates from 190 | 271 |
| 192 | 191 [FINAL: ARDS + Mechanical Ventilation + Etiology or Causation or Harm or Named Injuries; limits applied. Update period only. ] | 271 |

# CDSR

EBM Reviews - Cochrane Database of Systematic Reviews 2005 to August 16, 2023

| **#** | **Searches** | **Results** |
| --- | --- | --- |
| 1 | acute respiratory distress syndrome?.ti,ab. | 22 |
| 2 | adult respiratory distress syndrome?.ti,ab. | 0 |
| 3 | APRONet.ti,ab. | 0 |
| 4 | ARDS.ti,ab. | 18 |
| 5 | ARDSnet*.ti,ab. | 0 |
| 6 | ARDSS.ti,ab. | 0 |
| 7 | shock lung.ti,ab. | 0 |
| 8 | (acute adj1 chest adj1 syndrome?).ti,ab. | 15 |
| 9 | (acute adj1 lung? adj1 fail*).ti,ab. | 0 |
| 10 | (acute adj1 pulmonary adj1 fail*).ti,ab. | 0 |
| 11 | (acute adj1 bronchopulmonary adj1 fail*).ti,ab. | 0 |
| 12 | (acute adj1 respirat* adj1 fail*).ti,ab. | 9 |
| 13 | (acute adj1 lung? adj1 injur*).ti,ab. | 12 |
| 14 | (acute adj1 pulmonary adj1 injur*).ti,ab. | 1 |
| 15 | (acute adj1 bronchopulmonary adj1 injur*).ti,ab. | 0 |
| 16 | (acute adj1 respirat* adj1 injur*).ti,ab. | 0 |
| 17 | ALI.ti,ab. [ Acute Lung Injury ] | 4 |
| 18 | or/1-17 [ ARDS ] | 50 |
| 19 | (respirator or respirators).ti,ab. | 4 |
| 20 | (ventilator or ventilators).ti,ab. | 70 |
| 21 | (mechan* adj5 ventilat*).ti,ab. | 217 |
| 22 | (pressure? adj5 ventilat*).ti,ab. | 73 |
| 23 | (respirator* adj2 therap*).ti,ab. | 7 |
| 24 | (inhalat* adj2 therap*).ti,ab. | 1 |
| 25 | (oxygen* adj2 therap*).ti,ab. | 86 |
| 26 | ventilation.ti,ab. | 332 |
| 27 | ventilatory.ti,ab. | 41 |
| 28 | (art#ficial adj1 respiration).ti,ab. | 0 |
| 29 | (art#ficial adj1 breath*).ti,ab. | 0 |
| 30 | (ventilat* adj2 support*).ti,ab. | 41 |
| 31 | (respirator* adj2 support*).ti,ab. | 61 |
| 32 | (breath* adj3 support*).ti,ab. | 1 |
| 33 | continuous positive airway pressure?.ti,ab. | 64 |
| 34 | (hyperbaric adj oxygenat*).ti,ab. | 1 |
| 35 | (intermittent* adj1 positive-pressure?).ti,ab. | 26 |
| 36 | (positive-pressure adj1 respirat*).ti,ab. | 1 |
| 37 | cpap.ti,ab. | 53 |
| 38 | peep.ti,ab. | 9 |
| 39 | wean*3.ti,ab. | 36 |
| 40 | or/19-39 [ Mechanical Ventilation & related terms ] | 491 |
| 41 | 18 and 40 [ ARDS + Mechanical Ventilation ] | 25 |
| 42 | (adverse?? adj2 effect?).ti,ab. | 2060 |
| 43 | (adverse?? adj2 event?).ti,ab. | 3085 |
| 44 | (cause? or causing or causal* or causation).ti,ab. | 2456 |
| 45 | (harm or harms or harmed or harming or harmful*).ti,ab. | 2131 |
| 46 | cohort?.ti,ab. | 361 |
| 47 | complication?.ti,ab. | 1463 |
| 48 | death??.ti,ab. | 1538 |
| 49 | aetiolog*.ti,ab. | 119 |
| 50 | etiolog*.ti,ab. | 41 |
| 51 | iatrogen*.ti,ab. | 27 |
| 52 | morbidit*.ti,ab. | 1344 |
| 53 | mortalit*.ti,ab. | 2338 |
| 54 | odds ratio*.ti,ab. | 1042 |
| 55 | outcome?.ti,ab. | 7106 |
| 56 | risk*3.ti,ab. | 6994 |
| 57 | "health status indicat*".ti,ab. | 0 |
| 58 | "karnofsky performance".ti,ab. | 3 |
| 59 | "severity of illness*".ti,ab. | 11 |
| 60 | acute physiology scor*3.ti,ab. | 1 |
| 61 | (inpatient? adj2 acuit*).ti,ab. | 0 |
| 62 | (lung? adj3 collaps*).ti,ab. | 4 |
| 63 | (lung? adj4 iatrogen*).ti,ab. | 0 |
| 64 | (lung? adj4 injur*).ti,ab. | 31 |
| 65 | (myocardia* adj2 infarc*).ti,ab. | 211 |
| 66 | (organ? adj1 dysfunction adj1 scor*3).ti,ab. | 0 |
| 67 | (organ? adj2 fail*).ti,ab. | 24 |
| 68 | (patient? adj2 acuit*).ti,ab. | 2 |
| 69 | (pulmonary adj4 iatrogen*).ti,ab. | 0 |
| 70 | (pulmonary adj4 injur*).ti,ab. | 2 |
| 71 | sickness impact?.ti,ab. | 2 |
| 72 | (stroke or strokes).ti,ab. | 485 |
| 73 | (tracheal adj1 injur*).ti,ab. | 0 |
| 74 | (tracheal adj1 stenos#s).ti,ab. | 0 |
| 75 | (ventilator adj2 lung adj2 injur*).ti,ab. | 6 |
| 76 | APACHE.ti,ab. | 3 |
| 77 | arrhythmia?.ti,ab. | 71 |
| 78 | atelectas#s.ti,ab. | 14 |
| 79 | barotrauma*.ti,ab. | 15 |
| 80 | bleed*.ti,ab. | 499 |
| 81 | bronchopulmonary dysplasia*.ti,ab. | 67 |
| 82 | CVA.ti,ab. | 3 |
| 83 | deep venous thrombos#s.ti,ab. | 21 |
| 84 | diaphragm?.ti,ab. | 10 |
| 85 | DVT.ti,ab. | 52 |
| 86 | h?emorrhage?.ti,ab. | 330 |
| 87 | HLQoL.ti,ab. | 0 |
| 88 | HL-QoL.ti,ab. | 0 |
| 89 | morbidit*.ti,ab. | 1344 |
| 90 | mortalit*3.ti,ab. | 2338 |
| 91 | myocardial infarction?.ti,ab. | 209 |
| 92 | pneumomediastinum.ti,ab. | 1 |
| 93 | pneumonia?.ti,ab. | 213 |
| 94 | pneumopericardium.ti,ab. | 0 |
| 95 | pneumothoraces.ti,ab. | 4 |
| 96 | pneumothorax.ti,ab. | 60 |
| 97 | pulmonary emboli*.ti,ab. | 79 |
| 98 | QALI.ti,ab. | 0 |
| 99 | QALY.ti,ab. | 6 |
| 100 | QoL.ti,ab. | 186 |
| 101 | shock.ti,ab. | 61 |
| 102 | subcutaneous emphysema.ti,ab. | 1 |
| 103 | thromboemboli*.ti,ab. | 149 |
| 104 | troponin*.ti,ab. | 3 |
| 105 | ulcer?.ti,ab. | 187 |
| 106 | VAP.ti,ab. | 13 |
| 107 | VTE.ti,ab. | 49 |
| 108 | or/42-107 [ Etiology / Causation / Harm / Named Injuries ] | 9359 |
| 109 | 41 and 108 [ ARDS + Mechanical Ventilation + Etiology/Causation/Harm/Named Injuries ] | 24 |
| 110 | 109 not (adolescence or adolescent or adolescents or babies or baby or boy or boys or child or childhood or children or girl or girls or infancy or infant or infants or neonatal or neonatally or neonate or neonates or newborn or newborns or paediatric or paediatrician or paediatricians or paediatrics or pediatric or pediatrician or pediatricians or pediatrics or teen or teenage or teenagers or teens or youth or youths).ti,gw. | 20 |
| 111 | limit 110 to full systematic reviews | 20 |
| 112 | remove duplicates from 111 | 20 |
| 113 | limit 112 to last 4 years | 4 |

# ClinicalTrials.Gov

Acute Respiratory Distress Syndrome OR Acute Lung Injury OR Acute Chest Syndrome

AND

(complication* OR harm* OR etiology OR causation OR prognosis)

<https://classic.clinicaltrials.gov/ct2/results?cond=Acute+Respiratory+Distress+Syndrome+OR+Acute+Lung+Injury+OR+Acute+Chest+Syndrome&term=%28complication*+OR+harm*+OR+etiology+OR+causation+OR+prognosis%29&type=&rslt=With&recrs=d&recrs=g&recrs=h&recrs=e&recrs=i&recrs=m&age_v=&age=1&age=2&gndr=&intr=&titles=&outc=&spons=&lead=&id=&cntry=&state=&city=&dist=&locn=&rsub=&strd_s=06%2F01%2F2019&strd_e=08%2F17%2F2023&prcd_s=&prcd_e=&sfpd_s=&sfpd_e=&rfpd_s=&rfpd_e=&lupd_s=&lupd_e=&sort=>


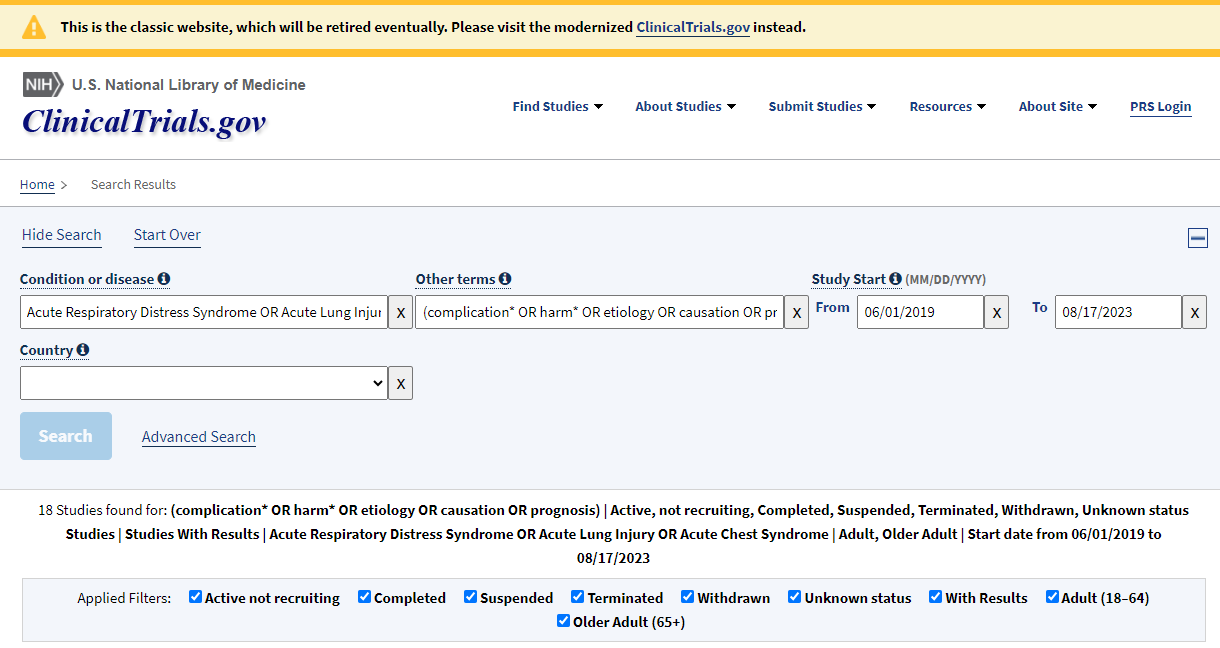

Supplement: Supplementary file 1 — Supplementary Material 1. [file 13054_2026_5978_MOESM1_ESM.docx]
