# Supplementary material for: Complications in acute respiratory distress syndrome: a systematic review and meta-analysis
Source: Crit Care. 2026 Mar 29;30:238. doi: 10.1186/s13054-026-05978-y (PMC13151369; doi:10.1186/s13054-026-05978-y)
Supplement: Supplementary file 2 — Supplementary Material 2. [file 13054_2026_5978_MOESM2_ESM.docx]

**Supplementary Materials**

**Supplementary methods**

**Supplementary results**

**eTable 1. Characteristics of randomized controlled trials**

**eTable 2. Characteristics of cohort studies**

**eTable 3. Etiology of ARDS in randomized controlled trials**

**eTable 4. Etiology of ARDS in cohort studies**

**eTable 5. Comorbidities of individuals with ARDS requiring IMV in randomized controlled trials**

**eTable 6. Other comorbidities of individuals with ARDS requiring IMV in randomized controlled trials**

**eTable 7. Comorbidities of individuals with ARDS requiring IMV in cohort studies**

**eTable 8. Other comorbidities of individuals with ARDS requiring IMV in cohort studies**

**eTable 9. Cochrane Risk of Bias-2 assessment of randomized controlled trials**

**eTable 10. Newcastle-Ottawa quality assessment for cohort studies with comparators**

**eTable 11. Newcastle-Ottawa quality assessment for cohort studies without comparators**

**eTable 12. Reported complications in randomized controlled trials not pooled in meta-analysis**

**eTable 13. Reported complications in cohort studies not pooled in meta-analysis**

**eFigure 1. Modified Newcastle Ottawa Scale**

**eFigure 2. Study identification**

**eFigure 3. Forest plot for barotrauma in cohort studies**

**eFigure 4. Forest plot for ventilator associated pneumonia in cohort studies**

**eFigure 5. Forest plot for acute renal failure in cohort studies**

**eFigure 6. Forest plot for sepsis in cohort studies**

**eFigure 7. Forest plot for bacteremia in cohort studies**

**eFigure 8. Forest plot for hospital mortality in cohort studies**

**eFigure 9. Forest plot for barotrauma in randomized controlled trials**

**eFigure 10. Forest plot for ventilator associated pneumonia in randomized controlled trials**

**eFigure 11. Forest plot for hypotension in randomized controlled trials**

**eFigure 12. Forest plot for arrhythmia in randomized controlled trials**

**eFigure 13. Forest plot for stroke in randomized controlled trials**

**eFigure 14. Forest plot for myopathy in randomized controlled trials**

**eFigure 15. Forest plot for cardiac arrest in randomized controlled trials**

**eFigure 16. Forest plot for hospital mortality in randomized controlled trials**

**References randomized controlled trials**

**References cohort studies**

**Supplementary methods**

Subgroup analyses

We performed a subgroup analysis comparing pooled complication prevalence between high and low risk of bias (ROB) studies. For cohort studies, any study not receiving full points on the Newcastle-Ottawa Scale was deemed high ROB. For randomized controlled trials, any trial rated as either “some concerns” or “high ROB” was deemed high ROB for subgroup analyses. Subgroup analyses were restricted to complications with at least three studies in each ROB category to allow for stability of within subgroup estimates. We fitted separate random-effects models within each subgroup Pooled prevalence estimates were calculated using a random effects model with restricted maximum likelihood estimations and logit transformations of proportions. Confidence intervals were calculated using the Hartung–Knapp–Sidik–Jonkman method. A continuity correction of 0.5 was applied when necessary. Subgroup differences were tested using meta-regression with risk of bias as a moderator.

**Supplementary Results**

Subgroup analyses

Cohort studies

Complications amenable to subgroup analysis included barotrauma, ventilator associated pneumonia and acute renal failure. In subgroup analyses comparing cohort studies at high versus low overall risk of bias, there was no evidence of a difference in pooled prevalence of barotrauma (F= 0.045, p = 0.84, residual heterogeneity τ² = 0.15; I² = 81%), ventilator associated pneumonia (F=0.046, p=0.83, residual heterogeneity τ² = 1.9; I² = 99.6%), and acute renal failure (F=2.59, p=0.15, residual heterogeneity τ² = 0.45; I² = 98.6%). Study quality explained no between study heterogeneity in barotrauma and ventilator associated pneumonia (R² = 0%) and explained 14.8% between study heterogeneity in acute renal failure (R² = 14.8%).

Randomized controlled trials

Complications amenable to subgroup analysis included barotrauma, ventilator associated pneumonia and arrhythmia. In subgroup analyses comparing randomized controlled trials at high versus low overall risk of bias, there was no evidence of a difference in pooled prevalence of barotrauma (F= 0.196, p = 0.67, residual heterogeneity τ² = 0.29; I² = 92.2%), ventilator associated pneumonia (F=0.64 p=0.47, residual heterogeneity τ² = 2.77; I² = 99.1%), and arrhythmia (F=1.36, p=0.3, residual heterogeneity τ² = 0.29; I² = 93%). Risk of bias explained no between study heterogeneity in barotrauma and ventilator associated pneumonia (R² = 0%) and explained 3.5% between study heterogeneity in arrhythmia (R² = 3.5%).

| **eTable 1: Characteristics of randomized controlled trials** | | | | | |
| --- | --- | --- | --- | --- | --- |
| **Author, year** | **Total participants** | **Mean age (years)** | **ARDS definition** | **Mean**  **PaO₂ / FiO₂** | **Complications** |
| Allam 2020 | 240 | 51.5 | Murray score >3 |  | Gastropathy  Myopathy |
| Anzueto 1996 | 725 | 63.3 | AECC | 142.5 | Barotrauma  VAP  GI bleed  Liver dysfunction  Hypotension  Arrhythmia  Acute renal failure  Worsening secretions  Worsening mental status  Extubation/displaced endotracheal tube |
| Barrot 2020 | 201 | 57.8 | Berlin | 118.5 | Barotrauma  VAP  GI bleed  Arrhythmia  Bacteremia  Stroke  Delirium  Bowel ischemia  Seizure  Hemoptysis |
| Beitler 2019 | 200 | 51.5 | Berlin | 92.6 | Barotrauma |
| Brower 2000 | 861 | 51.5 | AECC | 136.0 | Barotrauma |
| Brower 2004 | 549 | 50.9 | AECC | 158.0 | Barotrauma |
| Cavalcanti 2017 | 1010 | 62.0 | AECC | 118.3 | Barotrauma  Cardiac arrest |
| Constantin 2019 | 400 | 54.5 | Berlin |  | Barotrauma  VAP |
| Ferguson 2013 | 548 | 58.0 | AECC | 117.5 | Barotrauma |
| Gattinoni 2001 | 304 | 59.0 | AECC | 127.4 | Pressure Sore  Extubation/displaced endotracheal tube  Loss of venous access  Displaced chest tube |
| Guérin 2013 | 466 | 52.8 | AECC | 100.0 | Barotrauma  Cardiac arrest  Extubation/displaced endotracheal tube  Airway/Endotracheal tube obstruction  Hemoptysis |
| Kacmarek 2016 | 200 | 47.1 | AECC | 130.5 | Barotrauma  Hypotension  Arrhythmia  Cardiac arrest |
| Khan 2018 | 490 | 51.5 | Not described | 197.0 | Barotrauma |
| Matthay 2011 | 282 | 55.7 | AECC | 170.5 | Arrhythmia |
| Meade 2008 | 983 | 60.0 | AECC | 144.7 | Barotrauma |
| Mercat 2008 | 767 | 55.8 | AECC | 143.5 | Barotrauma |
| Moss 2019 | 1006 | 58.0 | Berlin | 99.1 | Barotrauma  VAP  Hypotension  Arrhythmia  Myocardial infarction  Stroke  Neuropathy  Myopathy  Weakness  Gastrointestinal dysmotility  Subarachnoid hemorrhage  Subdural hematoma  Seizure  Superficial venous thrombosis  Hematoma not specified  Airway/endotracheal tube obstruction  Aspiration  Methemoglobinemia  Retroperitoneal hemorrhage |
| Papazian 2010 | 339 | 52.0 | AECC | 110.3 | Barotrauma  Weakness |
| Rice 2012 | 1000 | 63.3 | AECC | 166.0 | VAP  Bacteremia  Gastrointestinal infection |
| Richard 2024 | 699 | 55.0 | Berlin | 134 | Barotrauma |
| Smith 2012 | 326 | 50.0 | AECC | 103.5 | Arrhythmia |
| Taylor 2004 | 385 | 54.2 | AECC | 135.5 | Barotrauma  VAP  Acute renal failure  Infection not specified |
| Truwit 2014 | 745 | 57.0 | AECC | 170.0 | Arrhythmia  Myocardial infarction  Stroke  Myopathy  Deep vein thrombosis  Hyperthermia  Bowel ischemia |
| Villar 2020 | 277 | 56.0 | Berlin | 142.9 | Barotrauma  VAP  Sepsis  Genitourinary infection  Hyperglycemia  Empyema  Tracheobronchitis |
| Zeiher 2004 | 487 | 51.5 | AECC | 148.7 | Infection not specified |

| **eTable 2: Characteristics of cohort studies** | | | | | |
| --- | --- | --- | --- | --- | --- |
| **Author, year** | **Total participants** | **Mean age (years)** | **ARDS definition** | **Mean**  **PaO₂ / FiO₂** | **Complications** |
| Azoulay 2018 | 4953 | N/A | Berlin | N/A | VAP |
| Boissier 2015 | 216 | 61.7 | Berlin | 121.3 | Barotrauma |
| Chaiwat 2016 | 238 | 62.1 | AECC | N/A | VAP  Gastrointestinal bleed  Acute renal failure  Sepsis  Deep vein thrombosis |
| Contou 2016 | 423 | 62.0 | Berlin | 106.0 | VAP  Invasive pulmonary aspergillosis |
| Dobry 2025 | 384 | 53.8 | Not described | 70.5 | Barotrauma  Hypotension  Arrhythmia  Seizure  Anaphylaxis  Bronchospasm |
| Eachempati 2007 | 343 | 65.4 | AECC | N/A | VAP |
| Gacouin 2020 | 572 | 58.1 | AECC | 89.1 | VAP |
| Henry 2021 | 1266 | 56.0 | Not described | N/A | VAP  Myocardial infarction  Acute renal failure  Sepsis  Skin and soft tissue infection  Bacteremia  Stroke  Deep vein thrombosis  Pulmonary embolism  Compartment syndrome |
| Hsu 2024 | 263 | 59.8 | Berlin | N/A | VAP  Bacteremia |
| Hu 2021 | 264 | 39.7 | Berlin | 149.6 | Bacteremia  Viremia |
| Huang 2020 | 400 | N/A | Berlin | N/A | Barotrauma |
| Jeong 2021 | 229 | 72.0 | Berlin | 110.9 | Acute renal failure  Sepsis |
| LePape 2022 | 235 | 66.0 | Berlin |  | VAP |
| Lhéritier 2013 | 201 | 57.0 | AECC | 115.0 | Barotrauma |
| Liu 2022 | 264 | 54.7 | Berlin | 123.9 | Barotrauma |
| McNicholas 2023 | 1957 | N/A | Berlin | N/A | Acute renal failure |
| Nin 2017 | 1899 | 56.8 | AECC | 175.4 | Barotrauma  VAP  Liver dysfunction  Cardiac dysfunction  Acute renal failure  Sepsis  Hematologic failure |
| Panitchote 2019 | 357 | 53.0 | Berlin | 138.3 | Acute renal failure |
| Rahimibashar 2022 | 4200 | 67.2 | Not described | N/A | Delirium |
| Ruan 2016 | 547 | 62.8 | Berlin | 112.3 | Acute renal failure |
| Schellongowski 2025 | 709 | 61.0 | Berlin | N/A | VAP  Sepsis  Infection not specified  Bleeding |
| Todur 2023 | 222 | 52.5 | Berlin | N/A | Acute renal failure |
| Tsai 2020 | 241 | 59.7 | Berlin | 85.2 | VAP  Bacteremia  Genitourinary infection |
| Villar 2013 | 282 | 56.0 | AECC | 112.0 | Barotrauma |
| Wang 2021 | 2138 | N/A | Berlin | N/A | Acute renal failure |
| Wu 2022 | 382 | N/A | Berlin | N/A | Barotrauma  Subcutaneous emphysema |
| Yoo 2020 | 228 | 70.6 | Berlin | N/A | Acute renal failure |
| Zampieri 2018 | 524 | 61.8 | Berlin | N/A | VAP  Ventilator-associated tracheobronchitis |

| **eTable 3: Etiology of ARDS in randomized controlled trials** | | | |
| --- | --- | --- | --- |
| **Etiology** | **No. studies** | **Total participants N** | **Frequency n (%)** |
| Sepsis | 20 | 11820 | 3193 (27.0) |
| Pneumonia | 18 | 10396 | 5960 (57.3) |
| Aspiration | 19 | 11095 | 1544 (13.9) |
| Trauma | 14 | 8197 | 813 (9.9) |
| Pancreatitis | 6 | 3462 | 148 (4.3) |
| Burns/smoke inhalation | 3 | 2806 | 45 (1.6) |
| Transfusion | 10 | 6828 | 244 (3.6) |
| Surgery | 3 | 1721 | 177 (10.3) |
| Drug-induced | 3 | 2319 | 45 (1.9) |
| Other | 19 | 11095 | 1109 (10.0) |
|  | | | |
| Pulmonary | 4 | 1430 | 956 (66.9) |
| Extrapulmonary | 4 | 1430 | 474 (33.1) |

| **eTable 4: Etiology of ARDS in cohort studies** | | | |
| --- | --- | --- | --- |
| **Etiology** | **No. studies** | **Total participants N** | **Frequency n (%)** |
| Sepsis | 13 | 4915 | 800 (16.3) |
| Pneumonia | 14 | 5076 | 3264 (64.3) |
| Aspiration | 10 | 3761 | 568 (15.1) |
| Trauma | 4 | 1273 | 358 (28.1) |
| Pancreatitis | 3 | 1283 | 32 (2.5) |
| Transfusion | 2 | 1272 | 61 (4.8) |
| Drug-induced | 3 | 1516 | 23 (1.5) |
| Other | 11 | 4135 | 744 (18.0) |
|  | | | |
| Pulmonary | 3 | 7313 | 5273 (72.1) |
| Extrapulmonary | 3 | 7313 | 2040 (27.9) |

| **eTable 5: Comorbidities of individuals with ARDS requiring IMV in randomized controlled trials** | | | |
| --- | --- | --- | --- |
| **Comorbidity** | **No. studies** | **Total participants N** | **Frequency n (%)** |
| Obesity | 1 | 1000 | 438 (43.8) |
| Cancer | 2 | 667 | 98 (14.7) |
| Immune suppression | 3 | 1006 | 369 (36.7) |
| Chronic liver disease | 2 | 667 | 43 (6.5) |
| Chronic kidney disease | 4 | 1812 | 56 (3.1) |
| Chronic lung disease | 3 | 1067 | 140 (13.1) |
| Cardiac disease | 1 | 466 | 48 (10.3) |
| Diabetes | 5 | 2694 | 641 (23.8) |

| **eTable 6: Other comorbidities of individuals with ARDS requiring IMV in randomized controlled trials** | | | |
| --- | --- | --- | --- |
| **Comorbidity** | **No. studies** | **Total participants N** | **Frequency n (%)** |
| Solid cancer | 1 | 400 | 5 (1.3) |
| Hematologic cancer | 2 | 400 | 16 (4.0) |
| Coronary artery disease | 1 | 201 | 13 (6.5) |
| Congestive heart failure | 1 | 201 | 14 (7.0) |

| **eTable 7: Comorbidities of individuals with ARDS requiring IMV in cohort studies** | | | |
| --- | --- | --- | --- |
| **Comorbidity** | **No. studies** | **Total participants N** | **Frequency n (%)** |
| Obesity | 2 | 835 | 278 (33.3) |
| Cancer | 8 | 8791 | 1372 (15.6) |
| Immune suppression | 5 | 1875 | 337 (18.0) |
| Chronic liver disease | 13 | 10807 | 747 (6.9) |
| Chronic kidney disease | 13 | 6788 | 508 (7.5) |
| Chronic lung disease | 11 | 9568 | 1617 (16.9) |
| Cardiac disease | 8 | 7241 | 1077 (14.9) |
| Neurologic disease | 10 | 4013 | 335 (8.3) |
| Diabetes | 14 | 7360 | 1509 (20.5) |

| **eTable 8: Other comorbidities of individuals with ARDS requiring IMV in cohort studies** | | | |
| --- | --- | --- | --- |
| **Comorbidity** | **No. studies** | **Total participants N** | **Frequency n (%)** |
| Alcohol use disorder | 3 | 2174 | 275 (12.7) |
| Non-metastatic cancer | 1 | 524 | 57 (10.9) |
| Metastatic cancer | 1 | 524 | 15 (2.9) |
| Hematologic cancer | 3 | 1780 | 625 (35.1) |
| HIV | 2 | 5477 | 111 (2.0) |
| Coronary artery disease | 2 | 1813 | 70 (3.9) |
| Congestive heart failure | 1 | 547 | 38 (6.9) |
| Obstructive lung disease | 1 | 547 | 58 (10.6) |
| Structural lung disease | 1 | 547 | 60 (11.0) |
| Solid cancer | 2 | 1256 | 360 (28.7) |
| Hypertension | 6 | 4548 | 1435 (31.6) |
| Chronic respiratory failure | 1 | 235 | 34 (14.5) |
| Hematopoietic stem cell transplant | 2 | 973 | 486 (49.9) |
| Cognitive impairment/dementia | 2 | 5466 | 753 (13.8) |
| Aplasia or recent chemotherapy | 1 | 572 | 98 (17.1) |
| Peripheral vascular disease | 1 | 1266 | 8 (0.6) |
| Graft versus host disease | 1 | 709 | 106 (15.0) |
| Non-COPD respiratory disorder | 1 | 235 | 32 (13.6) |
| Autoimmune disease | 1 | 263 | 18 (6.8) |
| Bleeding diathesis | 1 | 1266 | 111 (8.8) |
| Pulmonary hypertension | 1 | 384 | 7 (1.8) |

| **eTable 9: Cochrane Risk of Bias-2 assessment of randomized controlled trials** | | | | | | | | |
| --- | --- | --- | --- | --- | --- | --- | --- | --- |
|  | **Risk of Bias Domain** | | | | | | | |
| **Study** | **D1** | **D2** | **D3** | | **D4** | **D5** | **Overall** |  |
| Allam 2020 | 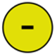 | 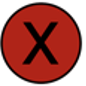 | 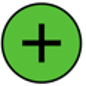 | | 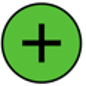 | 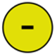 | 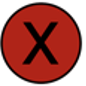 |  |
| Anzueto 1996 | 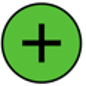 | 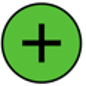 | 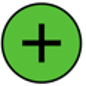 | | 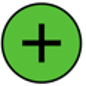 | 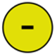 | 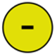 |  |
| Barrot 2020 | 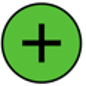 | 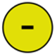 | 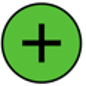 | | 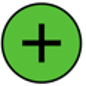 | 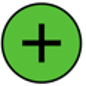 | 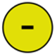 |  |
| Beitler 2019 | 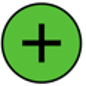 | 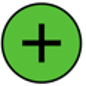 | 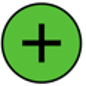 | | 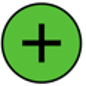 | 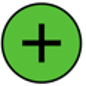 | 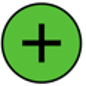 |  |
| Brower 2000 | 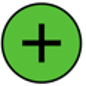 | 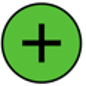 | 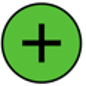 | | 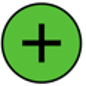 | 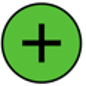 | 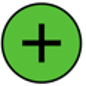 |  |
| Brower 2004 | 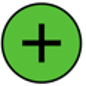 | 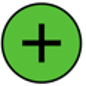 | 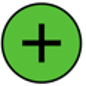 | | 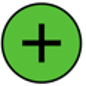 | 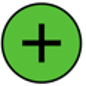 | 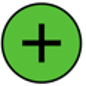 |  |
| Cavalcanti 2011 | 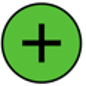 | 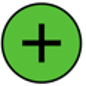 | 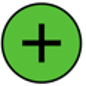 | | 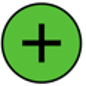 | 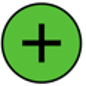 | 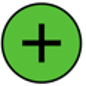 |  |
| Constantin 2019 | 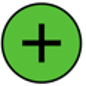 | 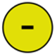 | 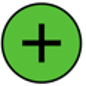 | | 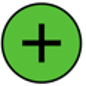 | 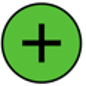 | 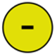 |  |
| Ferguson 2013 | 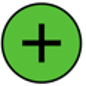 | 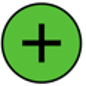 | 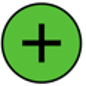 | | 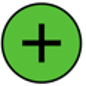 | 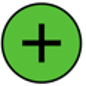 | 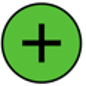 |  |
| Gattinoni 2001 | 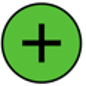 | 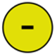 | 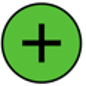 | | 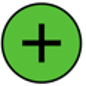 | 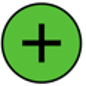 | 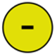 |  |
| Guérin 2013 | 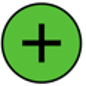 | 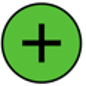 | 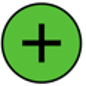 | | 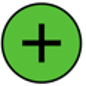 | 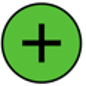 | 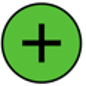 |  |
| Kacmarek 2016 | 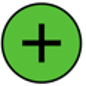 | 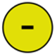 | 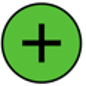 | | 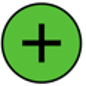 | 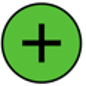 | 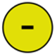 |  |
| Khan 2018 | 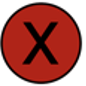 | 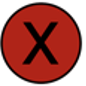 | 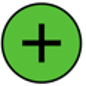 | | 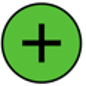 | 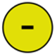 | 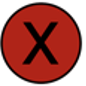 |  |
| Matthay 2011 | 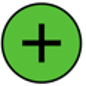 | 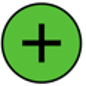 | 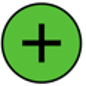 | | 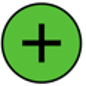 | 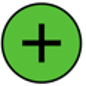 | 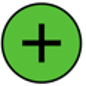 |  |
| Meade 2008 | 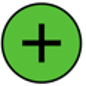 | 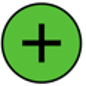 | 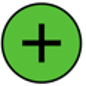 | | 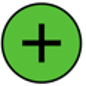 | 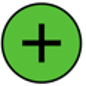 | 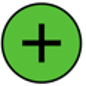 |  |
| Mercat 2008 | 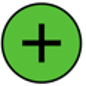 | 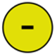 | 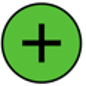 | | 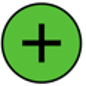 | 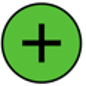 | 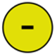 |  |
| Moss 2019 | 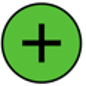 | 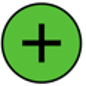 | 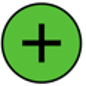 | | 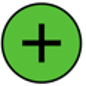 |  |  |  |
| Papazian 2010 |  |  |  | |  |  |  |  |
| Rice 2012 |  |  |  | |  |  |  |  |
| Richard 2024 |  |  |  | |  |  |  |  |
| Smith 2012 |  |  |  | |  |  |  |  |
| Taylor 2004 |  |  |  | |  |  |  |  |
| Truwit 2014 |  |  |  | |  |  |  |  |
| Villar 2020 |  |  |  | |  |  |  |  |
| Zeiher 2004 |  |  |  | |  |  |  |  |
| **Domains:**  D1: Bias arising from the randomization process  D2: Bias due to deviations from intended intervention  D3: Bias due to missing outcome data  D4: Bias in measurement of the outcome  D5: Bias in selection of the reported result | | | | **Judgement:**  High  Some concerns  Low | | | |  |

| **eTable 10: Newcastle-Ottawa quality assessment for cohort studies with comparators** | | | | |
| --- | --- | --- | --- | --- |
| **Study** | **Selection**  **(Max = 4)** | **Comparability**  **(Max = 2)** | **Outcome**  **(Max = 3)** | **Total**  **(Max = 9)** |
| Azoulay 2018 | 4 | 2 | 3 | 9 |
| Boissier 2015 | 4 | 0 | 3 | 7 |
| Chaiwat 2016 | 4 | 0 | 3 | 7 |
| Contou 2016 | 4 | 2 | 3 | 9 |
| Dobry 2025 | 3 | 2 | 3 | 8 |
| Gacouin 2020 | 4 | 2 | 3 | 9 |
| Henry 2021 | 3 | 2 | 3 | 8 |
| Hsu 2024 | 4 | 2 | 2 | 8 |
| Hu 2021 | 4 | 2 | 3 | 9 |
| Jeong 2021 | 4 | 2 | 3 | 9 |
| Le Pape 2022 | 4 | 2 | 3 | 9 |
| Lhéritier 2013 | 4 | 0 | 3 | 7 |
| Liu 2022 | 4 | 2 | 2 | 8 |
| McNicholas 2023 | 4 | 2 | 3 | 9 |
| Nin 2017 | 4 | 2 | 3 | 9 |
| Panitchote 2019 | 4 | 2 | 3 | 9 |
| Rahimibashar 2022 | 3 | 0 | 3 | 6 |
| Ruan 2016 | 4 | 2 | 3 | 9 |
| Tsai 2020 | 4 | 2 | 2 | 8 |
| Wang 2021 | 4 | 2 | 2 | 8 |
| Wu 2022 | 4 | 2 | 3 | 9 |
| Zampieri 2018 | 4 | 2 | 3 | 9 |

| **eTable 11: Newcastle-Ottawa quality assessment for cohort studies without comparators** | | | | |
| --- | --- | --- | --- | --- |
| **Study** | **Selection**  **(Max = 3)** | **Comparability**  **(N/A; Max = 0)** | **Outcome**  **(Max = 3)** | **Total**  **(Max = 6)** |
| Eachempati 2007 | 3 | 0 | 2 | 5 |
| Huang 2020 | 3 | 0 | 3 | 6 |
| Schellongowski 2025 | 3 | 0 | 3 | 6 |
| Todur 2023 | 3 | 0 | 3 | 6 |
| Villar 2013 | 3 | 0 | 3 | 6 |
| Yoo 2020 | 3 | 0 | 3 | 6 |

| **eTable 12: Reported complications in randomized controlled trials not pooled in meta-analysis** | | | |
| --- | --- | --- | --- |
| **Complication** | **No. studies** | **Total participants N** | **Frequency n (%)** |
| Gastrointestinal bleed | 2 | 926 | 37 (4.0) |
| Liver dysfunction | 1 | 725 | 40 (5.5) |
| Myocardial infarction | 2 | 1751 | 6 (0.3) |
| Acute kidney injury | 2 | 1110 | 96 (8.7) |
| Sepsis | 1 | 277 | 25 (9.0) |
| Bacteremia | 2 | 1201 | 135 (11.2) |
| Gastrointestinal infection | 1 | 1000 | 28 (2.8) |
| Genitourinary infection | 1 | 277 | 2 (0.7) |
| Infection not specified | 1 | 872 | 107 (12.3) |
| Neuropathy | 1 | 1006 | 1 (0.1) |
| Weakness | 2 | 1345 | 264 (19.6) |
| Delirium | 1 | 201 | 22 (10.9) |
| Deep vein thrombosis | 1 | 745 | 48 (6.4) |
| Gastrointestinal dysmotility | 1 | 1006 | 1 (0.1) |
| Hyperthermia | 1 | 745 | 3 (0.4) |
| Bowel ischemia | 2 | 946 | 17 (1.8) |
| Pressure sore | 1 | 304 | 97 (31.9) |
| Extubation/displaced endotracheal tube | 2 | 1029 | 45 (4.4) |
| Loss of venous access | 1 | 304 | 22 (7.2) |
| Displaced chest tube | 1 | 304 | 7 (2.3) |
| Worsening secretions | 1 | 725 | 131 (18.1) |
| Worsening mental status | 1 | 725 | 65 (9.0) |
| Hemoptysis | 2 | 667 | 25 (3.7) |
| Subarachnoid hemorrhage | 1 | 1006 | 1 (0.1) |
| Subdural hematoma | 1 | 1006 | 1 (0.1) |
| Seizure | 2 | 1207 | 3 (0.2) |
| Superficial venous thrombosis | 1 | 1006 | 1 (0.1) |
| Hematoma not specified | 1 | 1006 | 1 (0.1) |
| Retroperitoneal hemorrhage | 1 | 1006 | 1 (0.1) |
| Airway/endotracheal tube obstruction | 2 | 1472 | 17 (1.2) |
| Aspiration | 1 | 1006 | 2 (0.2) |
| Methemoglobinemia | 1 | 1006 | 2 (0.2) |
| Gastropathy | 1 | 240 | 50 (20.8) |
| Hyperglycemia | 1 | 277 | 202 (72.9) |
| Empyema | 1 | 277 | 2 (0.7) |
| Tracheobronchitis | 1 | 277 | 1 (0.4) |

| **eTable 13: Reported complications in cohort studies not pooled in meta-analysis** | | | |
| --- | --- | --- | --- |
| **Complication** | **No. studies** | **Total participants N** | **Frequency n (%)** |
| Gastrointestinal bleed | 1 | 238 | 4 (1.7) |
| Liver dysfunction | 1 | 1899 | 274 (14.4) |
| Hypotension | 1 | 384 | 248 (64.6) |
| Arrhythmia | 1 | 384 | 21 (5.5) |
| Myocardial infarction | 1 | 1266 | 32 (2.5) |
| Cardiac dysfunction | 1 | 1899 | 1285 (67.7) |
| Skin and soft tissue infection | 1 | 1266 | 36 (2.8) |
| Genitourinary infection | 1 | 241 | 18 (7.5) |
| Infection not specified | 1 | 709 | 208 (29.3) |
| Stroke | 1 | 1266 | 51 (4.0) |
| Delirium | 1 | 4200 | 1437 (34.2) |
| Deep vein thrombosis | 2 | 1504 | 140 (9.3) |
| Pulmonary embolism | 1 | 1266 | 51 (4.0) |
| Ventilator-associated tracheobronchitis | 1 | 524 | 54 (10.3) |
| Invasive pulmonary aspergillosis | 1 | 423 | 17 (4.0) |
| Hematologic failure | 1 | 1899 | 520 (27.4) |
| Viremia | 1 | 264 | 18 (6.8) |
| Compartment syndrome | 1 | 1266 | 13 (1.0) |
| Subcutaneous emphysema | 1 | 382 | 21 (5.5) |
| Seizure | 1 | 384 | 13 (3.4) |
| Anaphylaxis | 1 | 384 | 2 (0.5) |
| Bronchospasm | 1 | 384 | 2 (0.5) |
| Bleeding | 1 | 709 | 534 (75.3) |

**eFigure 1: Modified Newcastle Ottawa Scale**

1a) Representativeness of exposed cohort

- Truly representative (1 star)
- Somewhat representative (1 star)
- Selected group
- No description of derivation of cohort

1b) Selection of the non-exposed cohort

- Drawn from the same community as exposed cohort (1 star)
- Drawn from a different source
- No description of derivation of the non-exposed cohort
- N/A (ie no non-exposed cohort)

1c) Ascertainment of exposure

- Secure record (1 star)
- Structured interview (1 star)
- Written self report
- No description
- Other

1d) Demonstration that outcome (clinical complication) of interest was not present at the start of study

- Yes (1 star)
- No

2a) Comparability of the cohorts on the basis of the design or analysis controlled for cofounders

- The study controls for the most important factor (some metric of disease severity) (1 star)
- Study does not control for the above
- N/A (ie no non-exposed cohort)

2b) Comparability of the cohorts on the basis of the design or analysis controlled for cofounders

- The study controls for any additional factor (1 star)
- Study does not control for any additional factors
- N/A (ie no non-exposed cohort)

3a) Assessment of outcome

- Independent blind assessment (1 star)
- Record linkage (1 star)
- Self report
- No description
- Other

3b) Was follow up long enough for outcomes to occur

- Yes (1 star)
- No

3c) Adequacy of follow-up of cohorts

- Complete follow up (all subjects accounted for) (1 star)
- Subjects lost unlikely to introduce bias (number lost less than 20% or description provided of those lost) (1 star)
- Follow up rate less than 80% and no description of those lost
- No statement

**eFigure 2:** Study identification

**eFigure 3: Forest plot for barotrauma in cohort studies**

**eFigure 4: Forest plot for ventilator associated pneumonia in cohort studies**

**eFigure 5: Forest plot for acute renal failure in cohort studies**

**eFigure 6: Forest plot for sepsis in cohort studies**

**eFigure 7: Forest plot for bacteremia in cohort studies**

**eFigure 8: Forest plot for hospital mortality in cohort studies**

**eFigure 9: Forest plot for barotrauma in randomized controlled trials**

**eFigure 10: Forest plot for ventilator associated pneumonia in randomized controlled trials**

**eFigure 11: Forest plot for hypotension in randomized controlled trials**

**eFigure 12: Forest plot for arrhythmia in randomized controlled trials**

**eFigure 13: Forest plot for stroke in randomized controlled trials**

**eFigure 14: Forest plot for myopathy in randomized controlled trials**

**eFigure 15: Forest plot for cardiac arrest in randomized controlled trials**

**eFigure 16: Forest plot for hospital mortality in randomized controlled trials**

**References Randomized Control Trials:**

1. Allam M. Comparative study between the uses of high dose corticosteroid therapy for short duration versus low dose corticosteroid for long duration in severe lung contusion with ARDS. Open Anesthesia J. 2020;14:90–100. doi:10.2174/2589645802014010090.
2. Anzueto A, Baughman RP, Guntupalli KK, et al; Exosurf Acute Respiratory Distress Syndrome Sepsis Study Group. Aerosolized surfactant in adults with sepsis-induced acute respiratory distress syndrome.. N Engl J Med. 1996;334(22):1417-1421. doi:10.1056/NEJM199605303342201
3. Barrot L, Asfar P, Mauny F, et al. Liberal or conservative oxygen therapy for acute respiratory distress syndrome. N Engl J Med. 2020;382(11):999-1008. doi:10.1056/NEJMoa1916431
4. Beitler JR, Sarge T, Banner-Goodspeed VM, et al. Effect of titrating positive end-expiratory pressure (PEEP) with an esophageal pressure-guided strategy vs an empirical high PEEP-FiO2 strategy on death and days free from mechanical ventilation among patients with acute respiratory distress syndrome: A Randomized Clinical Trial. JAMA. 2019;321(9):846-857. doi:10.1001/jama.2019.0555
5. Brower RG, Matthay MA, Morris A, et al; Acute Respiratory Distress Syndrome Network. Ventilation with lower tidal volumes as compared with traditional tidal volumes for acute lung injury and the acute respiratory distress syndrome. N Engl J Med. 2000;342(18):1301-1308. doi:10.1056/NEJM200005043421801
6. Brower RG, Lanken PN, MacIntyre N, et al. Higher versus lower positive end-expiratory pressures in patients with the acute respiratory distress syndrome. N Engl J Med. 2004;351(4):327-336. doi:10.1056/NEJMoa032193
7. Cavalcanti AB, Suzumura ÉA, Laranjeira LN, et al; Writing Group for the Alveolar Recruitment for Acute Respiratory Distress Syndrome Trial (ART) Investigators. Effect of lung recruitment and titrated positive end-expiratory pressure (PEEP) vs low PEEP on mortality in patients with acute respiratory distress syndrome: A Randomized Clinical Trial. JAMA. 2017;318(14):1335-1345. doi:10.1001/jama.2017.14171
8. Constantin JM, Jabaudon M, Lefrant JY, et al. Personalised mechanical ventilation tailored to lung morphology versus low positive end-expiratory pressure for patients with acute respiratory distress syndrome in France (the LIVE study): a multicentre, single-blind, randomised controlled trial. Lancet Respir Med. 2019;7(10):870-880. doi:10.1016/S2213-2600(19)30138-9
9. Ferguson ND, Cook DJ, Guyatt GH, et al. High-frequency oscillation in early acute respiratory distress syndrome. N Engl J Med. 2013;368(9):795-805. doi:10.1056/NEJMoa1215554
10. Gattinoni L, Tognoni G, Pesenti A, et al. Effect of prone positioning on the survival of patients with acute respiratory failure. N Engl J Med. 2001;345(8):568-573. doi:10.1056/NEJMoa010043
11. Guérin C, Reignier J, Richard JC, et al. Prone positioning in severe acute respiratory distress syndrome. N Engl J Med. 2013;368(23):2159-2168. doi:10.1056/NEJMoa1214103
12. Kacmarek RM, Villar J, Sulemanji D, et al. Open Lung Approach for the Acute Respiratory Distress Syndrome: A Pilot, Randomized Controlled Trial. Crit Care Med. 2016;44(1):32-42. doi:10.1097/CCM.0000000000001383
13. Khan A. Is the lung recruitment and titrated positive end expiratory pressure a better strategy as compare to low PEEP on mortality in patients with acute respiratory distress syndrome. Medical Forum Monthly. 2018;29(04)
14. Matthay MA, Brower RG, Carson S, et al; National Heart, Lung, and Blood Institute Acute Respiratory Distress Syndrome (ARDS) Clinical Trials Network. Randomized, placebo-controlled clinical trial of an aerosolized β₂-agonist for treatment of acute lung injury. Am J Respir Crit Care Med. 2011;184(5):561-568. doi:10.1164/rccm.201012-2090OC
15. Meade MO, Cook DJ, Guyatt GH, et al. Ventilation strategy using low tidal volumes, recruitment maneuvers, and high positive end-expiratory pressure for acute lung injury and acute respiratory distress syndrome: a randomized controlled trial. JAMA. 2008;299(6):637-645. doi:10.1001/jama.299.6.637
16. Mercat A, Richard JC, Vielle B, et al. Positive end-expiratory pressure setting in adults with acute lung injury and acute respiratory distress syndrome: a randomized controlled trial. JAMA. 2008;299(6):646-655. doi:10.1001/jama.299.6.646
17. Moss M, Huang DT, Brower RG, et al; National Heart, Lung, and Blood Institute PETAL Clinical Trials Network. Early neuromuscular blockade in the acute respiratory distress syndrome. N Engl J Med. 2019;380(21):1997-2008. doi:10.1056/NEJMoa1901686
18. Papazian L, Forel JM, Gacouin A, et al. Neuromuscular blockers in early acute respiratory distress syndrome. N Engl J Med. 2010;363(12):1107-1116. doi:10.1056/NEJMoa1005372
19. Rice TW, Wheeler AP, Thompson BT et al; National Heart, Lung, and Blood Institute Acute Respiratory Distress Syndrome (ARDS) Clinical Trials Network. Initial trophic vs full enteral feeding in patients with acute lung injury: the EDEN randomized trial. JAMA. 2012;307(8):795-803. doi:10.1001/jama.2012.137
20. Richard JM, Beloncle FM, Béduneau G, et al. Pressure control plus spontaneous ventilation versus volume assist-control ventilation in acute respiratory distress syndrome. A randomised clinical trial. Intensive Care Med. 2024;50(10):1647-1656. doi:10.1007/s00134-024-07612-3
21. Smith FG, Perkins GD, Gates S, et al. Effect of intravenous β-2 agonist treatment on clinical outcomes in acute respiratory distress syndrome (BALTI-2): a multicentre, randomised controlled trial. Lancet. 2012;379(9812):229-235. doi:10.1016/S0140-6736(11)61623-1
22. Taylor RW, Zimmerman JL, Dellinger RP, et al. Low-dose inhaled nitric oxide in patients with acute lung injury: a randomized controlled trial. JAMA. 2004;291(13):1603-1609. doi:10.1001/jama.291.13.1603
23. Truwit JD, Bernard GR, Steingrub J et al; National Heart, Lung, and Blood Institute ARDS Clinical Trials Network. Rosuvastatin for sepsis-associated acute respiratory distress syndrome. N Engl J Med. 2014;370(23):2191-2200. doi:10.1056/NEJMoa1401520
24. Villar J, Ferrando C, Martínez D, et al. Dexamethasone treatment for the acute respiratory distress syndrome: a multicentre, randomised controlled trial. Lancet Respir Med. 2020;8(3):267-276. doi:10.1016/S2213-2600(19)30417-5
25. Zeiher BG, Artigas A, Vincent JL, et al. Neutrophil elastase inhibition in acute lung injury: results of the STRIVE study. Crit Care Med. 2004;32(8):1695-1702. doi:10.1097/01.ccm.0000133332.48386.85

**References Cohort Studies:**

1. Azoulay E, Lemiale V, Mourvillier B, et al. Management and outcomes of acute respiratory distress syndrome patients with and without comorbid conditions. Intensive Care Med. 2018;44(7):1050-1060. doi:10.1007/s00134-018-5209-6
2. Boissier F, Razazi K, Thille AW, et al. Echocardiographic detection of transpulmonary bubble transit during acute respiratory distress syndrome. Ann Intensive Care. 2015;5:5. doi:10.1186/s13613-015-0046-z
3. Chaiwat O, Chittawatanarat K, Piriyapathsom A, et al. Incidence of and Risk Factors for Acute Respiratory Distress Syndrome in Patients Admitted to Surgical Intensive Care Units: The Multicenter Thai University- Based Surgical Intensive Care Unit (THAI-SICU) Study. J Med Assoc Thai. 2016;99 Suppl 6:S118-S127.
4. Contou D, Dorison M, Rosman J, et al. Aspergillus-positive lower respiratory tract samples in patients with the acute respiratory distress syndrome: a 10-year retrospective study. Ann Intensive Care. 2016;6(1):52. doi:10.1186/s13613-016-0156-2
5. Dobry P, Lane R, Whittaker P, et al. Two paths to paralysis: A multicenter comparison of cisatracurium to atracurium in the management of acute respiratory distress syndrome. J Crit Care. 2026;91:155227. doi:10.1016/j.jcrc.2025.155227
6. Eachempati SR, Hydo LJ, Shou J, Barie PS. Outcomes of acute respiratory distress syndrome (ARDS) in elderly patients. J Trauma. 2007;63(2):344-350. doi:10.1097/TA.0b013e3180eea5a1
7. Gacouin A, Lesouhaitier M, Reizine F, et al. Short-term survival of acute respiratory distress syndrome patients due to influenza virus infection alone: a cohort study. ERJ Open Res. 2020;6(4):00587-2020. doi:10.1183/23120541.00587-2020
8. Henry R, Ghafil C, Piccinini A, et al. Extracorporeal support for trauma: A trauma quality improvement project (TQIP) analysis in patients with acute respiratory distress syndrome. Am J Emerg Med. 2021;48:170-176. doi:10.1016/j.ajem.2021.04.083
9. Hsu PC, Lin YT, Kao KC, et al. Risk factors for prolonged mechanical ventilation in critically ill patients with influenza-related acute respiratory distress syndrome. Respir Res. 2024;25(1):9. Published 2024 Jan 4. doi:10.1186/s12931-023-02648-3
10. Hu Y, Shen J, An Y, Liu S. Early high dose corticosteroid therapy in hematopoietic stem cell transplantation patients with acute respiratory distress syndrome: a propensity score matched study. Ther Adv Respir Dis. 2021;15:17534666211009397. doi:10.1177/17534666211009397
11. Huang X, Zhang R, Fan G, et al. Incidence and outcomes of acute respiratory distress syndrome in intensive care units of mainland China: a multicentre prospective longitudinal study. Crit Care. 2020;24(1):515. doi:10.1186/s13054-020-03112-0
12. Jeong JH, Heo M, Ju S, et al. Pulmonary mycobacterial infection is associated with increased mortality in patients with acute respiratory distress syndrome. Medicine (Baltimore). 2021;100(33):e26969. doi:10.1097/MD.0000000000026969
13. Le Pape M, Besnard C, Acatrinei C, et al. Clinical impact of ventilator-associated pneumonia in patients with the acute respiratory distress syndrome: a retrospective cohort study. Ann Intensive Care. 2022;12(1):24. doi:10.1186/s13613-022-00998-7
14. Lhéritier G, Legras A, Caille A, et al. Prevalence and prognostic value of acute cor pulmonale and patent foramen ovale in ventilated patients with early acute respiratory distress syndrome: a multicenter study. Intensive Care Med. 2013;39(10):1734-1742. doi:10.1007/s00134-013-3017-6
15. Liu X, Liu H, Liu S, et al. Effects of prone positioning for patients with acute respiratory distress syndrome caused by pulmonary contusion: a single-center retrospective Study. Can Respir J. 2022;2022:4579030. Published 2022 Mar 31. doi:10.1155/2022/4579030
16. McNicholas BA, Rezoagli E, Simpkin AJ, et al. Epidemiology and outcomes of early-onset AKI in COVID-19-related ARDS in comparison with non-COVID-19-related ARDS: insights from two prospective global cohort studies. Crit Care. 2023;27(1):3. doi:10.1186/s13054-022-04294-5
17. Nin N, Muriel A, Peñuelas O, et al. Severe hypercapnia and outcome of mechanically ventilated patients with moderate or severe acute respiratory distress syndrome. Intensive Care Med. 2017;43(2):200-208. doi:10.1007/s00134-016-4611-1
18. Panitchote A, Mehkri O, Hastings A, et al. Factors associated with acute kidney injury in acute respiratory distress syndrome. Ann Intensive Care. 2019;9(1):74. Published 2019 Jul 1. doi:10.1186/s13613-019-0552-5
19. Rahimibashar F, Miller AC, Salesi M, et al. Risk factors, time to onset and recurrence of delirium in a mixed medical-surgical ICU population: A secondary analysis using Cox and CHAID decision tree modeling. EXCLI J. 2022;21:30-46. Published 2022 Jan 4. doi:10.17179/excli2021-4381
20. Ruan SY, Wu HY, Lin HH, Wu HD, Yu CJ, Lai MS. Inhaled nitric oxide and the risk of renal dysfunction in patients with acute respiratory distress syndrome: a propensity-matched cohort study. Crit Care. 2016;20(1):389. doi:10.1186/s13054-016-1566-0
21. Schellongowski P, Darmon M, Eller P, et al. Acute respiratory distress syndrome in patients with cancer: the YELENNA prospective multinational observational cohort study. Intensive Care Med. 2025;51(10):1809-1819. doi:10.1007/s00134-025-08113-7
22. Todur P, Nileshwar A, Chaudhuri S, Srinivas T. Incidence, outcomes, and predictors of subphenotypes of acute kidney injury among acute respiratory distress syndrome patients: a prospective observational study. Indian J Crit Care Med. 2023;27(10):724-731. doi:10.5005/jp-journals-10071-24553
23. Tsai MJ, Yang KY, Chan MC, et al. Impact of corticosteroid treatment on clinical outcomes of influenza-associated ARDS: a nationwide multicenter study. Ann Intensive Care. 2020;10(1):26. doi:10.1186/s13613-020-0642-4
24. Villar J, Pérez-Méndez L, Blanco J, et al. A universal definition of ARDS: the PaO2/FiO2 ratio under a standard ventilatory setting--a prospective, multicenter validation study. Intensive Care Med. 2013;39(4):583-592. doi:10.1007/s00134-012-2803-x
25. Wang Y, Zhang L, Xi X, Zhou JX; China Critical Care Sepsis Trial (CCCST) Workgroup. the association between etiologies and mortality in acute respiratory distress syndrome: a multicenter observational cohort study. Front Med. 2021;8:739596. doi:10.3389/fmed.2021.739596
26. Wu SH, Kor CT, Li CY, Hsiao YC. Intermediate tidal volume is an acceptable option for ventilated patients with acute respiratory distress syndrome. Med Intensiva. 2022;46(11):609-618. doi:10.1016/j.medine.2022.03.002
27. Yoo JW, Ju S, Lee SJ, Cho YJ, Lee JD, Kim HC. Red cell distribution width/albumin ratio is associated with 60-day mortality in patients with acute respiratory distress syndrome. Infect Dis. 2020;52(4):266-270. doi:10.1080/23744235.2020.1717599
28. Zampieri FG, Póvoa P, Salluh JI, et al. Lower respiratory tract infection and short-term outcome in patients with acute respiratory distress syndrome. J Intensive Care Med. 2020;35(6):588-594. doi:10.1177/0885066618772498
